# Vacuum-assisted vertical component distribution in pseudo-bulk heterojunctions: a pathway to high-performance and stable organic solar cells

**DOI:** 10.1093/nsr/nwaf440

**Published:** 2025-10-20

**Authors:** Jingchao Cheng, Liang Wang, Tiantian Wang, Chen Chen, Yuandong Sun, Jing Zhou, Zirui Gan, Weiyi Xia, Dawei Gao, Dan Liu, Wei Li, Tao Wang

**Affiliations:** School of Materials and Microelectronics, Wuhan University of Technology, Wuhan 430070, China; School of Materials Science and Engineering, Wuhan University of Technology, Wuhan 430070, China; School of Materials Science and Engineering, Wuhan University of Technology, Wuhan 430070, China; School of Materials Science and Engineering, Wuhan University of Technology, Wuhan 430070, China; School of Materials Science and Engineering, Wuhan University of Technology, Wuhan 430070, China; School of Materials Science and Engineering, Wuhan University of Technology, Wuhan 430070, China; School of Materials Science and Engineering, Wuhan University of Technology, Wuhan 430070, China; School of Materials Science and Engineering, Wuhan University of Technology, Wuhan 430070, China; School of Materials and Microelectronics, Wuhan University of Technology, Wuhan 430070, China; School of Materials Science and Engineering, Wuhan University of Technology, Wuhan 430070, China; School of Materials Science and Engineering, Wuhan University of Technology, Wuhan 430070, China; State Key Laboratory of Advanced Glass Materials, Wuhan University of Technology, Wuhan 430070, China; School of Materials and Microelectronics, Wuhan University of Technology, Wuhan 430070, China

**Keywords:** organic solar cells, pseudo-bulk heterojunctions, vertical phase distribution, stability, morphology

## Abstract

Precise control over the vertical component distribution and molecular packing within the photoactive layer is paramount for achieving high-performance organic solar cells (OSCs). However, optimizing these parameters in sequentially deposited pseudo-bulk heterojunction (p-BHJ) architecture remains challenging, often limited by inefficient self-assembly of organic semiconductors. In this work, a post-treatment combining vacuum and thermal annealing (VTA) is adopted. A range of morphological measurements demonstrate that compared with the traditional thermal annealing (TA) treatment, VTA can accelerate solvent evaporation, promoting the crystallization of acceptor at the top surface and inhibiting excessive intermixing between donor and acceptor, and therefore leads to a more stratified p-i-n configuration with interpenetrated donor–acceptor networks. This morphology modulation enhances carrier mobility and suppresses charge recombination, culminating in a champion power conversion efficiency (PCE) of 20.5% for binary OSCs. Furthermore, due to the enhanced crystallinity and more compact molecular packing, the optimized film also exhibits exceptional photo and morphological stability, achieving a *T*_80_ of over 3900 hours in a conventional ITO/PEDOT:PSS/PM6/L8-BO/PDINN/Ag structure, and 54 000 h in an interfacial-stable ITO/MoO_3_/PM6/L8-BO/C_60_/BCP/Ag structure, under the continuous one-sun illumination with a criterion of ISOS-L-1.

## Teaser

Synergistic vacuum and thermal annealing orchestrate the vertical phase distribution in organic solar cells. This precise morphology modulation enhances crystallinity and eliminates free volume, leading to improved efficiency and operational stability.

## INTRODUCTION

Driven by escalating global demand for clean and sustainable energy, solar photovoltaics (PV) stands out as a foremost technology [1,2]. Among various PV technologies, organic solar cells (OSCs) exhibit promising prospects due to their advantages of light weight, flexibility, solution-processability and potential for large-area manufacturing [3–7]. Despite remarkable progress in power conversion efficiency (PCE) of OSCs exceeding 20% [8–13], the operational stability of OSCs, particularly under combined stressors including heat, light and humidity, remains a hurdle impeding their commercial viability [14–18].

Among various functional layers of OSCs, the photoactive layer governs exciton dissociation and charge transport [19–21], and plays a critical role in determining the operational stability of OSCs [14,22–24]. Conventional bulk heterojunction (BHJ) photoactive layers, formed via spontaneous phase separation of donor and acceptor, and blending during solution processing, often exhibit kinetically trapped nanostructures (e.g. free volume) that are susceptible to arousing morphology evolution of OSCs upon operation [24]. This metastable morphology triggers rapid performance degradation during initial operation, known as the burn-in process [25–28]. Planar heterojunctions (PHJs), achieved by stratifying donor/acceptor phases via orthogonal solvent processing, could enhance the operational stability of OSCs by suppressing molecular interdiffusion. However, the molecular weight, chemical structure and compatibility of organic semiconductors in PHJs should be rationally designed, to ensure efficient exciton dissociation and charge collection [29–31]. As such, construction of quasi-PHJs (Q-PHJs) or pseudo-BHJs (p-BHJs) through sequential deposition of donor and acceptor has aroused great interest [30–33]. This configuration establishes continuous carrier transport pathways along the vertical structure of OSCs via compositional gradients (donor/acceptor enrichment at the top and bottom surface of the photoactive layer) [34], and has demonstrated record efficiency (over 20% PCE) with robust stability (*T*_80_ > 1000 h) in OSCs [35,36].

In addition to the routinely used post-treatment like thermal annealing (TA) or solvent vapor annealing (SVA), vacuum-assisted post-treatment has also emerged as a practical way to tune the organization behavior of materials upon solution-processing [37–41]. For example, Hou and Marsal *et al.* revealed that the extra vacuum treatment upon film-forming of the BHJ photoactive layer could remove residual solvent and facilitate molecular crystallization, leading to improved light absorption and durability [37–39]. Additionally, by utilizing a vacuum following the TA treatment, Zhu *et al.* demonstrated that the sharp surface of the donor and acceptor in the PHJ photoactive layer processed from the orthogonal solvent could be suppressed, leading to a more intermixed phase with enhanced exciton dissociation [40,41]. However, despite the above advances, the understanding and application of vacuum treatment are still far less than for the conventional TA and SVA treatments, especially regarding their feasibility in controlling the molecular packing of donor and acceptor in the vertical direction of OSCs, which is of great importance for carrier collection upon device operation [30,31,40,41].

Herein, a post-treatment utilizing vacuum and thermal annealing (VTA) was concurrently employed to further mediate the vertical morphology of OSCs. We demonstrate that VTA can accelerate solvent evaporation kinetics, enhancing acceptor crystallization at the top interface. Furthermore, the formation of an excessively miscible phase was mitigated, therefore establishing a more stratified p-i-n configuration. This morphology modification further leads to more ordered molecular packing throughout the photoactive layer, which is beneficial for enhanced and balanced charge transport and operational stability. As a result, a remarkable PCE of 20.5% was achieved in D18/L8-BO OSCs. More importantly, the improved crystallinity and suppressed free volume elevate the photo and morphological stability of photovoltaic film, leading to a *T*_80_ of over 3900 h with a criterion of ISOS-L-1, and 54 000 h for the stable ITO/MoO_3_/PM6/L8-BO/C_60_/BCP/Ag structure. A *T*_80_ of over 1200 h was also demonstrated with a criterion of ISOS-L-3 [65°C ± 5°C, 65% ± 10% relative humidity (RH), one-sun illumination], under a conventional structure of ITO/PEDOT:PSS/PM6/L8-BO/PDINN/Ag.

## RESULTS AND DISCUSSION

**Figure 1.**
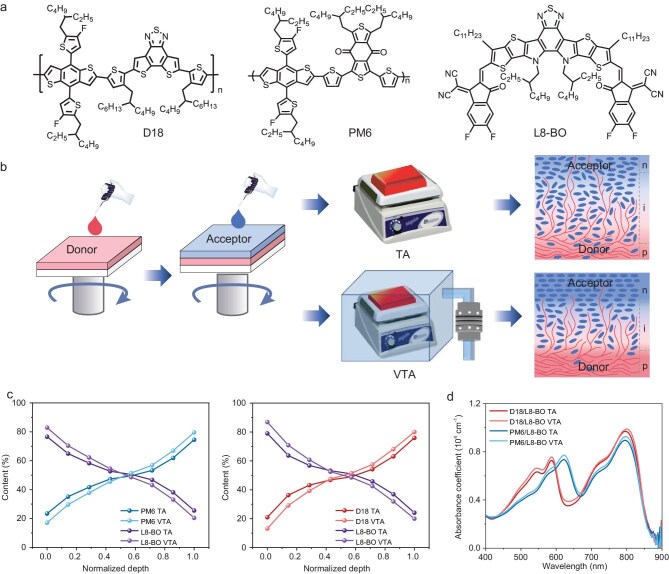
(a) Chemical structures of D18, PM6 and L8-BO. (b) Schematic illustration of TA and VTA treatments in p-BHJ. (c) Depth profiles from XPS measurements of D18/L8-BO and PM6/L8-BO films. (d) Absorption coefficient spectra of PM6/L8-BO and D18/L8-BO films upon different treatments.

The chemical structures of polymer donors (PM6, D18) and the non-fullerene acceptor (L8-BO) are shown in Fig. 1a. p-BHJ films were fabricated by sequentially casting the donor and acceptor layers. In addition to as-cast and routinely used TA treatment, VTA treatment was processed via transferring the as-cast film onto a preheated hot stage within an environment rapidly evacuated to low pressure (100 kPa vacuum) (a schematic illustration is shown in Fig. 1b and the detailed fabrication process is shown in the Supplementary data).

During the post-treatment, TA could activate the donor and acceptor molecules in the as-cast film and encourage their intermixing. Distinctly, the vacuum environment in VTA accelerates solvent volatilization from bottom to top, which could boost the crystallization of acceptor molecules near the top surface in the as-cast film, thereby facilitating the formation of a gradient p-i-n architecture. From depth-profiling X-ray photoelectron spectroscopy (XPS) analysis (Fig. 1c), it is clear to see that compared with the conventional TA treatment, VTA treatment induced an increased acceptor content at the top surface and an increased donor content at the bottom surface, suggesting more stratified composition distribution. From the further absorption spectra shown in Fig. 1d (with neat films shown in Fig. S1), VTA-treated films exhibit increased absorbance for polymer donors, and red shifts for non-fullerene acceptor compared with those in TA-treated films, implying enhanced structural order.

**Figure 2.**
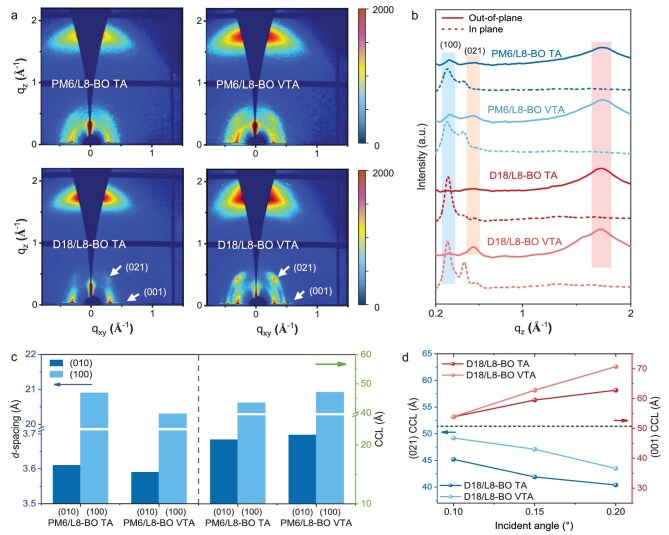
(a) 2D and (b) 1D GIWAXS profiles and (c) CCL and *d*-spacing values of corresponding films. (d) CCLs of (021) and (001) diffraction peaks in corresponding films versus varied incident angles.

Grazing-incidence wide-angle X-ray scattering (GIWAXS) was then performed, with the 2D pattern and 1D profiles shown in Fig. 2a and b (detailed GIWAXS data are shown in Table S1). Conventional TA-processed PM6/L8-BO film exhibits a preferential ‘face-on’ molecular orientation featuring an (100) lamellar ordering at *q*_z_ = 0.30 Å^−1^ in the in-plane (IP) and an (010) π–π stacking at *q*_z_ = 1.74 Å^−1^ in the out-of-plane (OOP) direction. For VTA-treated PM6/L8-BO film, a similar face-on molecular orientation with red-shifted (100) lamellar and (010) π–π diffraction peak at 0.31 and 1.75 Å^−1^ (corresponds to shortened distance from 3.61 and 20.9 Å to 3.59 and 20.3 Å) are observed, suggesting more compact molecular stacking. Meanwhile, intensified diffraction intensities, with increased (010) crystal coherence lengths (CCLs) from 20.9 to 21.7 Å and (100) CCLs from 43.5 to 47.1 Å (shown in Fig. 2c) are also observed, suggesting increased crystallinity. Additionally, for the D18/L8-BO film, VTA treatment not only delivered significantly intensified (11–1) and (021) diffraction peaks for L8-BO, but also enhanced the (001) diffraction peak for D18 at *q*_xy_ = 0.57 Å^−1^, suggesting improved molecular ordering for both donor and acceptor (Figs S2 and S3; Tables S2–S4). Such enhanced crystallinity and more compact structure are conducive to carrier transport and collection within corresponding OSCs.

Angle-dependent GIWAXS measurements were further performed to investigate vertical crystallization (Figs S4–S7) [42,43]. GIWAXS analysis of neat films (Figs S2 and S3; Tables S2–S4) reveals that the (001) diffraction peak at *q*_xy_ = 0.54–0.56 Å⁻^1^ originates from the lamellar stacking of D18, whereas the (021) peak at *q*_z_ = 0.52–0.54 Å⁻^1^ is attributed to backbone stacking of L8-BO. Selected incidence angles of 0.10°, 0.15° and 0.20° were utilized to probe the crystallographic information of photovoltaic film from near the top surface to the entire film (shown in Fig. 2d; Tables S5 and S6). Depth-profiling CCL analysis of the (021) diffraction peak reveals maximal CCLs at α = 0.10° (below the critical angle and therefore probing the top surface region of the film [43]) for the D18/L8-BO film, confirming the enhanced crystallinity of L8-BO near the top surface. With the incident angle increased to 0.15° and 0.20° (at or above the critical angle and therefore probing the whole film), the CCL of the (001) diffraction peak of D18 started to increase, which might be attributed to the synergistic effects of improved crystallinity and increased content of D18 near the bottom surface, in line with the XPS measurements shown earlier. Although it is difficult to deconvolute the PM6 and L8-BO phases due to their overlap within the diffraction peaks of (010) and (100), enhanced intensity for both (010) and (100) are observed throughout the active layer (shown in Fig. S8; Tables S7 and S8), confirming enhanced crystallinity by VTA. Combining the above observation for XPS and GIWAXS, we propose that the vacuum environment accelerates the solvent evaporation, which promotes the crystallization for the acceptor at the top surface and hampers the over-intermixing between donor and acceptor, leading to a vertical p-i-n configuration with interpenetrated donor–acceptor networks.

**Figure 3.**
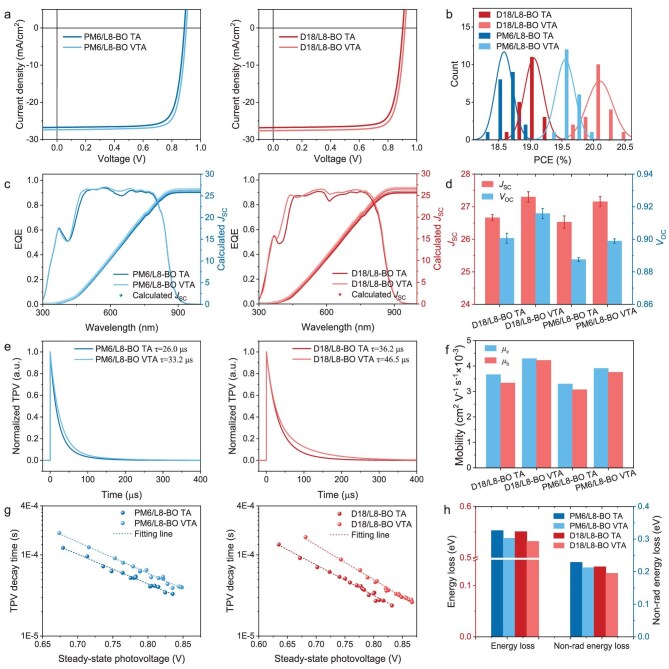
(a) *J–V* curves, (b) histogram of PCE counts from 20 devices, (c) EQE curves, (d) histogram of *J*_SC_ and *V*_OC_, (e) normalized TPV curves, (f) carrier mobilities, (g) steady-state TPV and (h) histogram of energy loss of corresponding OSCs.

OSCs were then fabricated using the above techniques, with their current density–voltage (*J–V*) characteristics and statistical performance shown in Fig. 3a and Table 1 (with device optimization details shown in Fig. S9; Tables S9 and S10). Conventional TA-processed devices using PM6/L8-BO and D18/L8-BO photoactive layers obtained maximum PCEs of 18.9% and 19.3%, accompanied by fill factors (FFs) of 79.4% and 80.1%, short-circuit current densities (*J*_SC_) of 26.9 and 26.8 mA cm⁻^2^, and open-circuit voltages (*V*_OC_) of 0.889 and 0.905 V, aligning with previous work [11,44]. Remarkably, VTA treatment elevated the device performance, achieving maximum PCEs of 20.0% (PM6/L8-BO) and 20.5% (D18/L8-BO), with significantly enhanced FF values approaching 81%, *J*_SC_ values of around 27.5 mA cm⁻^2^, and *V*_OC_ values of 0.902 and 0.922 V, respectively (Fig. 3b, Fig. S10 and Fig. 3d). Furthermore, when an all-polymer PM6/PY-IT system was processed with VTA, enhanced performance from 18.4% (TA-treated device) to 19.5% was obtained (Fig. S11 and Table S11), underscoring its versatility in tuning the morphology of p-BHJ-based photoactive layers. As shown in the external quantum efficiency (EQE) spectra in Fig. 3c, VTA-treated OSCs displayed intensified spectral response at the whole absorption range, suggesting that the charge generation and collection processes have been improved. This might be attributed to the synergistic effects of optimized vertical phase distribution and enhanced molecular order, facilitating charge transport and collection dynamics. The <5% discrepancy between *J*_SC_ values calculated from EQE and *J–V* measurements further validate the reliability.

**Table 1.** Photovoltaic parameters of different devices. Statistics of PCE, FF, *J*_sc_ and PCE were calculated from 20 individual devices.

Devices	PCE (%)	FF (%)	*J*_SC_ (mA·cm^−2^)	*J*_SC_^calculated^ (mA·cm^−2^)	*V*_OC_ (V)
PM6/L8-BO TA	18.9 (18.6 ± 0.2)	79.4 (78.9 ± 0.3)	26.9 (26.5 ± 0.2)	26.0	0.889 (0.888 ± 0.001)
PM6/L8-BO VTA	20.0 (19.6 ± 0.2)	80.9(80.2 ± 0.3)	27.5 (27.2 ± 0.2)	26.5	0.902 (0.899 ± 0.002)
D18/L8-BO TA	19.3 (19.1 ± 0.2)	80.1 (79.4 ± 0.4)	26.8 (26.7 ± 0.1)	25.8	0.905 (0.901 ± 0.003)
D18/L8-BO VTA	20.5 (20.1 ± 0.2)	80.9 (80.4 ± 0.4)	27.6 (27.3 ± 0.2)	26.7	0.922 (0.915 ± 0.003)

Furthermore, carrier lifetime and mobility of these devices were investigated through transient photocurrent (TPC) and space-charge-limited current (SCLC) measurements. As shown in Fig. 3e, VTA-processed devices extended charge carrier lifetime from 26.0 to 33.2 μs in PM6/L8-BO devices and from 36.2 to 46.5 μs in D18/L8-BO devices, demonstrating suppressed recombination. Concomitantly, improved and balanced charge mobility (Fig. 3f, Fig. S12 and Table S12) were also observed, which explains their superior FF and *J*_SC_. These improved carrier dynamics further lead to enhanced carrier collection and exciton dissociation efficiencies, as shown in Figs S13 and S14 and Table S13.

Given the enhanced *V*_OC_ of VTA-treated devices shown in Table 1, energy loss (*E*_loss_) quantification was further conducted, with results shown in Fig. 3h, Fig. S15 and Table S14. It can be seen that compared with the relatively large *E*_loss_ of 0.553 eV (PM6/L8-BO) and 0.551 eV (D18/L8-BO) in TA-processed devices, these values decreased to 0.538 and 0.532 eV in VTA-processed devices, respectively. In particular, non-radiative energy losses (Δ*E*_nr_) were notably reduced in VTA-treated devices, from 0.229 and 0.215 eV to 0.212 and 0.195 eV for the PM6-based and D18-based devices, confirming the effective suppression of non-radiative recombination. Steady-state transient photovoltage (TPV) analyses were further utilized to investigate carrier recombination in devices processed with different treatments. As shown in Fig. 3g and Fig. S16, prolonged carrier lifetimes are obtained across the photovoltages under different illumination intensity, demonstrating suppressed non-radiative recombination losses of VTA-based OSCs compared to TA controls. This suppression of carrier recombination can be attributed to the enhanced molecular ordering and percolated charge-conducting pathways, thereby elevating *J*_SC_ and *V*_OC_ [45].

**Figure 4.**
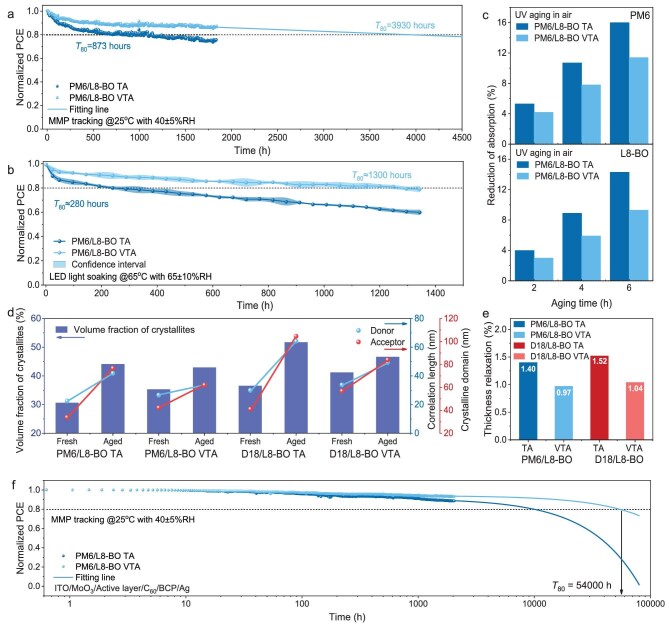
Normalized PCE of PM6/L8-BO OSCs processed with corresponding treatments under (a) ISOS-L-1 protocol and (b) ISOS-L-3 protocol. (c) Histogram of reduced absorption through UV aging, (d) fitting results of morphology parameters extracted from GISAXS profile and (e) normalized reduced thickness during isothermal annealing for corresponding films. (f) Normalized PCE of ITO/MoO_3_/PM6/L8-BO/C_60_/BCP/Ag OSCs processed with corresponding treatment under ISOS-L-1 protocol.

The operational stability of the OSCs was first evaluated under ISOS-L-1 protocols (continuous one-sun illumination, 25°C ± 5°C, 40% ± 5% RH) using maximum power point (MPP) tracking (Fig. 4a; Figs S17 and S18). Upon TA treatment, PM6/L8-BO-based devices exhibited a mild degradation in *J*_SC_ (around 1%), but suffered strong FF and *V*_OC_ decays of 12.6% and 12.5% after 1800 h, implying significantly increased recombination. In contrast, VTA-processed devices not only maintained a mild degradation of their *J*_SC_, like TA, but also suppressed FF and *V*_OC_ decay from 12.7% and 12.4% to 6.7% and 6.1%, allowing an extrapolated *T*_80_ of 3930 h. Additionally, for the D18/L8-BO system, significant *J*_SC_ decay of 5.4%, together with FF and *V*_OC_ decay of 15.3% and 14.7% shown in the TA-treated device upon operation for 1800 h, attributed to phase demixing of highly crystalized D18 reported in previous work [39]. By comparison, the VTA-processed devices suppressed the degradation, limiting *J*_SC_, FF and *V*_OC_ decay to only 1.1%, 8.5% and 8.8%, respectively, which corresponds to an extrapolated *T*_80_ lifetime to 2900 h.

Further stability validation under accelerated ISOS-L-3 protocols (65°C ± 5°C, 65% ± 10% RH, one-sun illumination) was conducted (Fig. 4b; Figs S19 and S20). The TA-processed devices suffered catastrophic performance decay of PCE of 37.4% (PM6/L8-BO) and 48.6% (D18/L8-BO) after 1200 h aging, accompanied by a collapse in *T*_80_ lifetimes to only 280 and 144 h, respectively. In striking contrast, the VTA-engineered devices demonstrated exceptional resilience, maintaining a restricted decay of 18.5% (PM6/L8-BO) and 23.8% (D18/L8-BO) after equivalent aging, leading to extended *T*_80_ lifetimes of 1300 and 960 h. The above stability measurements under ISOS-L-1 and ISOS-L-3 protocols confirm that VTA significantly enhances the operational stability of OSCs under combined thermal, light or moisture stresses, which might be attributed to the suppressed intermixing phase that kinetically retards phase segregation [27,29].

We noticed that VTA treatment effectively suppresses the burn-in of OSCs in the first 200 h, which is crucial to realize highly stable OSCs. To elucidate the underlying mechanism, UV photodegradation of PM6/L8-BO and D18/L8-BO films was conducted in ambient air. The UV aged absorption spectra (Fig. 4c and Fig. S21) reveal severe degradation in TA films, where the acceptor L8-BO experienced a more pronounced reduction in its absorbance across the PM6/L8-BO (15.0%) and D18/L8-BO (14.3%) films. In contrast, VTA treatment significantly reduced the acceptor decay to 9.3% (PM6-based) and 9.9% (D18-based). Additionally, it has been reported that benzodithiophene-thiophene (BDT-T) units in donor polymers (PM6, D18) are more vulnerable to photo-oxidative degradation [16]. Nevertheless, owing to the enhanced molecular order by VTA, the resulting PM6/L8-BO and D18/L8-BO films also exhibited decreased photo bleaching for the donor PM6 and D18 from 16.0% to 11.0% (PM6) and 15.4% to 10.8% (D18), compared with the values of the TA-treated film. These results collectively confirm that VTA enhances the overall photostability of the photoactive layer.

The morphological variations of the above films were then investigated. As atomic force microscopy (AFM) height images show in Fig. S22, the TA-processed films exhibit increased roughness from 1.0 to 1.8 nm in PM6/L8-BO and from 1.0 to 4.6 nm in D18/L8-BO, with enlarged aggregates, after thermal aging at 150°C for 4 h, while the VTA-processed films only show increased roughness on their surface, suggesting enhanced morphological stability. Grazing-incidence small-angle X-ray scattering (GISAXS) further revealed domain size evolution of the photoactive layer after thermal aging at 150°C for 4 h (Figs S23 and S24). As the simulated results show in Fig. 4d, and Tables S15 and S16, the TA-processed PM6/L8-BO film shows a coarsening of PM6 domain size from 22.5 to 41.8 nm and expansion of L8-BO crystalline domain from 33.9 to 76.7 nm. In contrast, suppressed domain size growth from 26.6 to 33.7 nm (PM6) and from 42.3 to 62.0 nm (L8-BO) was obtained. This trend was also observed in the D18/L8-BO system, demonstrating more robust morphological stability upon thermal stress.

*In situ* spectroscopic ellipsometry (Figs S25 and S26) was then employed to probe the origin of improved morphological stability of the VTA-processed film via evaluating its thermal relaxation behavior. As shown in Fig. 4e, the TA-processed PM6/L8-BO and D18/L8-BO films exhibit substantial thickness relaxation (>1.4%) after 30 min of thermal relaxation (at 150°C in an N_2_ atmosphere), whereas the VTA treatment suppresses relaxation decay to merely 0.97% (PM6-based) and 1.04% (D18-based). This attenuated relaxation correlates with the tighter molecular packing shown in the GIWAXS, and demonstrates that the VTA treatment is effective to suppress morphological traps (e.g. free volume) in the photoactive layer film, which can be the origin of the enhanced morphological stability. To further elucidate the role of the vacuum process (simplified as VA), the device performance and film characteristics of VA-treated OSCs are summarized in Fig. S27 and Table S17. Compared with TA- and VTA-processed films, the VA-processed film shows the lowest absorption coefficient and the strongest phase relaxation, suggesting that although VA could partially remove residual solvent, it could not drive the molecules to organize in an orderly manner. As shown by the results, an inferior photovoltaic performance of PCE of 18.6% is achieved in the VA-treated OSC, underscoring the synergistic effect of VA and TA to promote the crystallization and stratification of donor and acceptor in the vertical direction of the OSC, and retard the phase relaxation upon operation.

Finally, as shown in Fig. 4f, Figs S28 and S29, and Table S18, OSCs were fabricated with a highly stable ITO/MoO_3_/PM6/L8-BO/C_60_/BCP/Ag structure. This configuration minimizes the interfacial stability interference, thereby more effectively isolating the intrinsic stability of the active layer. Under the ISOS-L-1 standard MPP tracking, the device achieved an exceptional *T*_80_ lifetime of 54 000 h, outperforming the previously reported BHJ structured OSC with VTA treatment [39]. This result underscore the critical role of the VTA method in advancing p-BHJ photovoltaics.

## CONCLUSION

In conclusion, we developed a VTA post-treatment for manipulating the morphology of sequentially deposited p-BHJ photoactive layers. From a range of morphological characterizations including XPS depth profiling, AFM, GIWAXS, GISAXS and *in situ* ellipsometry, we revealed that VTA promotes the crystallization of both the acceptor and donor while suppressing their excessive intermixing, leading to a more stratified p-i-n configuration with interpenetrated donor–acceptor networks. This optimized morphology enhances carrier mobility and suppresses charge recombination, culminating in a champion PCE of 20.5% and 20% for D18/L8-BO and PM6/L8-BO OSCs. Furthermore, the enhanced crystallinity and more compact molecular packing establish exceptional photo and morphological stability, achieving *T*_80_ over 3900 h in a conventional ITO/PEDOT:PSS/PM6/L8-BO/PDINN/Ag structure, and 54 000 h in a stable ITO/MoO_3_/PM6/L8-BO/C_60_/BCP/Ag structure, under the ISOS-L-1 protocol. Moreover, under harsher testing conditions of ISOS-L-3 (65°C ± 5°C, 65% ± 10% RH, one-sun illumination), *T*_80_ over 1200 h is also achieved, confirming the resilience of VTA-processed film against various stresses including thermal, light and moisture.

## Supplementary Material

nwaf440_Supplemental_File
