# Supplementary material for: Vacuum-assisted vertical component distribution in pseudo-bulk heterojunctions: a pathway to high-performance and stable organic solar cells
Source: Natl Sci Rev. 2025 Oct 20;12(12):nwaf440. doi: 10.1093/nsr/nwaf440 (PMC12648560; doi:10.1093/nsr/nwaf440)
Supplement: nwaf440_Supplemental_File [file nwaf440_supplemental_file.pdf]

Supplementary Information

Vacuum Assisted Vertical Distribution in Pseudo-Bulk Heterojunctions: A Pathway to High-Performance and Stable Organic Solar Cells

Jingchao Cheng[1], Liang Wang[2], Tiantian Wang[2], Chen Chen[2], Yuandong Sun[2], Jing Zhou[2], Zirui Gan[2], Weiyi Xia[2], Dawei Gao[1], Dan Liu[2], Wei Li[2,3*], Tao Wang[1]

[1]School of Materials and Microelectronics, Wuhan University of Technology, Wuhan 430070, China

[2]School of Materials Science and Engineering, Wuhan University of Technology, Wuhan 430070, China

[3]State key Laboratory of Advanced Glass Materials, Wuhan University of Technology, Wuhan, 430070

*Corresponding author

E-mail: liwei1992@whut.edu.cn

Experimental

Materials

D18 (M_w = 110 kDa, PDI = 2.1), PM6 (M_w = 97781 Da, PDI = 2.4), L8-BO and PDINN were purchased from Solarmer Materials (Beijing) Inc. Fullerene (C_{60}) and 2,9-Dimethyl-4,7-diphenyl-1,10-phenanthroline (BCP) were purchased from Nanjing Zhiyan Technology Co., Ltd. PEDOT:PSS (4083) was purchased from CleviosTM. ZnO precursor solution was synthesized according to a previous literature report [1]. High-transmittance ITO-glass substrates (resistance ~10 Ω sq^{-1}, maximum transmittance ~94% at ~550 nm, size of 20 × 15 × 0.7 mm^3) were purchased from You Xuan Ltd. China.

Organic Solar cell fabrication.

Organic solar cells (OSCs) were fabricated with two different structures, where one was ITO/PEDOT:PSS/active layer/PDINN/Ag, and the other was ITO/MoO_3/active layer/C_{60}/BCP/Ag.

For ITO/PEDOT:PSS/active layer/PDINN/Ag structure, the pre-patterned ITO-glass substrates were cleaned by ultra-sonication in water, ethanol, and isopropyl alcohol for 15 minutes each. Then these glass/ITO substrates were further treated with ultraviolet/Ozone for another 15 minutes after drying at 100 °C on a hotplate to remove any organic contaminants. 20 nm PEDOT:PSS films were spin-coated at 5000 rpm on top of the cleaned glass/ITO substrates, following with thermal annealing at 150 °C for 10 minutes in ambient. For the as-cast active layer film, D18 and PM6 were firstly spin-coated from 4 and 6 mg/ml CF solution at 2000 rpm for 30 s, and the acceptor layer L8-BO were deposited on the donor layer from an 8 mg/ml CF solution at 2300 rpm for 30 s, and the optimal thicknesses of the donor layer and active layer were about 46 and 100 nm (with thickness error bar of ca. 2 nm), respectively. The as-cast film was then thermally annealed at 85 °C for 5 min for TA. While the as-cast film was promptly transferred onto a hot stage preheated to 85°C and rapidly evacuated to 100 kPa at a pumping rate of 20 kPa/s, followed at this pressure for 5 minutes under continuous vacuum for vacuum thermal annealing (VTA). 1.2 mg/mL PDINN solution was spin-coated at 3000 rpm onto the active layer to afford 10 nm electron transport layer. Finally, 100 nm Ag were thermally evaporated through a shadow mask under high vacuum to form the anode. The size of the OSCs defined by the overlapping of anode and cathode is 6.625 mm^2. For ITO/MoO_3/active layer/C_{60}/BCP/Ag structure, the pre-patterned ITO-glass substrates were cleaned as the same way and the active layers were processed as the same above. 10 nm MoO_3 was thermally evaporated through a shadow mask under a high vacuum. 10 nm C_{60} and 10 nm BCP were thermally evaporated through a shadow mask under high vacuum in sequence. Finally, 100 nm Ag were evaporated through the same way as former.

Characterizations.

Absorption spectra of films were carried out using a UV-visible spectrophotometer (HITACHI, Japan). The current density-voltage (*J-V*) measurements were performed under AM 1.5G (100 mW cm^{-2}) using a Newport 3A solar simulator (Newport, USA) in air at room temperature after the light intensity was calibrated using a standard silicon reference cell certified by the National Renewable Energy Laboratory (NREL, USA). *J-V* characteristics were measured using software developed by Ossila Ltd. (UK) together with a source meter unit (2612B, Keithley, USA). External quantum efficiency (EQE) was measured with an EQE system (Zolix, China) equipped with a standard Si diode. The surface morphology of active layer films was characterized by an atomic force microscope (AFM)

(Solver Next, NT-MDT, Russia) with probes using ETALON Series HA-NC (Scansens GmbH, Ostec Group, Germany). Grazing-incidence wide-angle X-ray scattering (GIWAXS) and grazing-incidence small-angle X-ray scattering (GISAXS) were conducted using the beamline BL02U2 and BL16B1 at the Shanghai Synchrotron Radiation Facility in China

XPS and depth profile measurements

XPS measurements were performed on the Kratos AXIS SUPRA using 4 kV cathode biased Al Ka radiative source. The base pressure in the analysis chamber was about 5×10^{-10} mbar. Depth profiling tests were conducted by using an Ar^+ sputtering gun operated at 1 keV with the raster size of 1 mm×1 mm. On the basis of the F/N atom ratio, the acceptor weight content ($A_{wt\%}$) at different depths can be calculated by the formulas:

$$A_{wt\%} = \frac{A_{wt}}{D_{wt}+A_{wt}} = \frac{n_A M_A}{n_A M_A + n_D M_D} = \frac{1}{1+\frac{n_D M_D}{n_A M_A}} \qquad 1$$

$$\frac{F}{N} = \frac{4\times n_A + 2\times n_D}{8\times n_A} \Rightarrow \frac{n_D}{n_A} = \frac{4F}{N} - 2 \quad \text{(PM6)} \qquad 2$$

$$\frac{F}{N} = \frac{4\times n_A + 2\times n_D}{8\times n_A + 2\times n_D} \Rightarrow \frac{n_D}{n_A} = \frac{2F}{N-F} - 2 \quad \text{(D18)} \qquad 3$$

$$A_{wt\%} = \frac{1}{1+\left(\frac{M_D}{M_A}\right)\left(\frac{F}{N}\right)} \qquad 4$$

M_A is the molecular weight of L8-BO, M_D is the molecular weight repeat unit of donor, n_A is the mole number of L8-BO, n_D is the mole number of the repeat units of donor, F/N is the atom ratio of F/N. The F/N atom ratios were calculated by the integration of areas of corresponding XPS peaks and the atomic sensitivity factors. The simplified equation is F/N atom ratio = (F peak area/F sensitivity factor)/(N peak area/N sensitivity factor).

SCLC measurements

The hole and electron mobilities of all devices were obtained by using the space charge limited current (SCLC) method, the electron-only device with the structure of ITO/ZnO/Active layer/PDINN/Ag and the hole-only device with the structure of ITO/PEDOT:PSS/Active layer/MoO_3/Ag were fabricated. The SCLC measurements employ the Mott-Gurney equation: $J = 9\varepsilon_0\varepsilon_r\mu V^2/8L^3$ to estimate the electron and hole mobilities from dark *J-V* curves obtained from these devices. Here, J is the current density, ε_r is the relative dielectric constant of the active layer, ε_0 is the permittivity of free space, μ is the

charge mobility, and L is the thickness of the active layer. $V = V_{app}$-V_{bi}, where V_{app} is the voltage applied to the OSC device, and V_{bi} is the built-in potential voltage.

Exciton and carrier dynamics measurements

Exciton dissociation efficiency and charge collection efficiency measurements: J_{ph} is defined as $J_{ph} = J_{light} - J_{dark}$, where J_{light} and J_{dark} are the photocurrent densities under illumination and in the dark, respectively. V_{eff} is defined as $V_{eff} = V_0 - V_a$, where V_0 is the voltage when J_{ph} is 0, and V_a is the applied voltage. J_{ph} corresponding to the maximum V_{eff} is defined as the saturation current (J_{sat}) value. Exciton dissociation efficiency (P_{diss}) is the J_{ph}/J_{sat} value under short circuit condition, and charge collection efficiency (P_{coll}) is the J_{ph}/J_{sat} value under maximum power output condition.

Transient photovoltage (TPV) and transient photocurrent (TPC) measurements

The charge extraction time and carrier lifetime were extracted from the fitting line of the TPC/TPV signal with the equation: y=a × e^(-x/τ) + c, where a is a constant that fits the peak high, x is time, τ corresponds to the charge extraction time (TPC)/carrier lifetime (TPV). The background illumination was supplied by a standard LED light source, while the pulsed light was generated by an arbitrary waveform generator. The photovoltage signals were recorded using an oscilloscope, and the photocurrent traces were measured with a 50 Ω resistor, switching between open-circuit and short-circuit configurations. The integrated TPC signal provides a measure of the total charge generated by the laser pulse (ΔQ). V_{loss} consists of radiative (ΔV_r) and non-radiative recombination (ΔV_{nr}). The radiative decay rate of the charge transfer CT states (k_r) determines ΔV_r, the ratio of k_r and the non-radiative decay rate of CT states (k_{nr}) determines ΔV_{nr}. As the voltage decay time (the lifetime of the photo-generated charge carriers) is closely related to the inverse of CT state decay rate: $k_r + k_{nr} \approx k_{nr}$, the k_{nr} can be characterize by transient photovoltage decay (TPV) measurements.[2]

Non-radiative energy loss calculation

The $E_{loss}^{non\text{-}rad}$ is calculated through the following equation $E_{loss}^{non\text{-}rad} = V_{OC}^{rad} - V_{OC}$, in which V_{OC}^{rad} was carried out by formula:

$$V_{OC}^{rad} = \frac{kT}{q}\ln\left(\frac{J_{SC}}{J_0^{rad}} + 1\right) \cong \frac{kT}{q}\ln\left(1 + \frac{q\cdot\int_0^{+\infty} EQE(E)\cdot\emptyset_{AM\,1.5G}(E)\cdot dE}{q\cdot\int_0^{+\infty} EQE(E)\cdot\emptyset_{BB}(E)\cdot dE}\right) \quad 5$$

where the k is Boltzmann's constant, T is the temperature of the solar cell (T = 300 K is used in this paper), q is elementary charge, $\emptyset_{AM1.5G}(E)$ and $\emptyset_{BB}(E)$ are the standard solar spectrum under

AM1.5G (100 mW cm^{-2}) and black body spectrum at the temperature T of the solar cell, respectively.

GISAXS modelling

1D GISAXS profiles were fitted by using a Debye-Anderson-Brumberger (DAB)+Fractal model expressed in formula 6 via fitting software SASView (Version 4.2.2). The first term of formula 6 assigned to DAB is used for simulating the scattering of polymer domain, in which ξ is the average correlation length, q is the scattering wave vector, and A_1 is an independent fitting parameter. The second term of the formula is assigned to Fractal model, which means the occupation of fractal-like structure of the non-fullerene acceptor. P(q, R) and S(q, R) are the form factor and fractal structure factor, respectively. P(q, R) includes the product of acceptor particle volume fraction ϕ, the square of scattering length density difference between crystalline and amorphous $\Delta\rho^2$ and the crystalline particle volume V_P. S(q) is given by formula 7, where ϕ refers to the relative volume fraction of crystallites, $2R$ refers to the size of the primary acceptor crystalline particle. The correlation length and the fractal dimension of the fractal-like acceptor aggregates are represented by η and D, respectively. Guinier radius (R_g) is used to characterize the average domain size of acceptor phase (see formula 8).[3]

$$\mathrm{I(q)} = \frac{A_1}{[1+(q\xi)^2]^2} + A_2\big(P(q,R)\big)S(q,R,\eta,D) + B \qquad 6$$

$$S(q) = 1 + \frac{\sin[(D-1)\tan^{-1}(q\eta)]}{(qR)^D}\frac{D\Gamma(D-1)}{\left[1+\frac{1}{(q\eta)^2}\right]^{(D-1)/2}} \qquad 7$$

$$R_g = \sqrt{\frac{D(D+1)}{2}}\eta \qquad 8$$

In-situ ellipsometry measurements

Film thickness was measured through ellipsometry using the Cauchy model. The roughness was first fitted during iso-hold and then the roughness value was fixed during the global fitting of thickness, which substantially reduces the deviations during fitting (Figure S17). The transparent range from 950 to 1000 nm was selected to perform the thickness and roughness fitting. All films were coated on silicon substrate. For each system, three films were utilized to verify the effectiveness of measurements. For relaxation behavior measurement, samples were in-situ heated in a chamber filled with N_2, and the film thickness was collected at the initial temperature of 25 °C, with the ramp rate

of 25 °C min^{-1}. Once the temperature has reached 150 °C, this temperature was kept constant for 30 mins to study structural relaxation.

Operational stability characterizations of solar cells

The operational stability of devices was measured by maximum power point (MPP) tracking mode. The decay curves of these encapsulated devices were measured under continuous LED light source (400-900 nm, one-sun intensity) in ambient air conditions (the relative humidity was 40±5%, the tested temperature was 25±5 °C) for ISOS-L-1 protocol. For the decay curves measured under ISOS-L-3 protocol, a continuous LED light source (400-900 nm, one-sun intensity) in the relative humidity of 65±10% with temperature of 65±5 °C were processed and controlled by integrating thermocouple, humidity controller in a sealed box. For the device encapsulation, a layer of UV-curable adhesive was first smeared on the surface of device in the N_2 atmosphere, and a coverslip was put on the top following with UV illumination for 1 min. For the UV-aging of films, 365 nm UV light (20 mW cm^{-2}) was incident from the top side (acceptor side) of the film, and the variation of their absorption spectra were recorded.

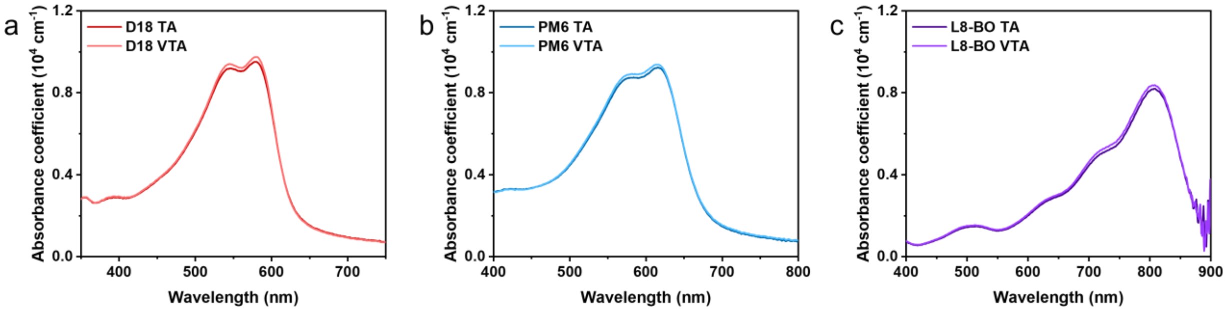

Figure S1. Absorption coefficient spectra of (a) D18, (b) PM6 and (c) L8-BO films fabricated upon corresponding treatments.

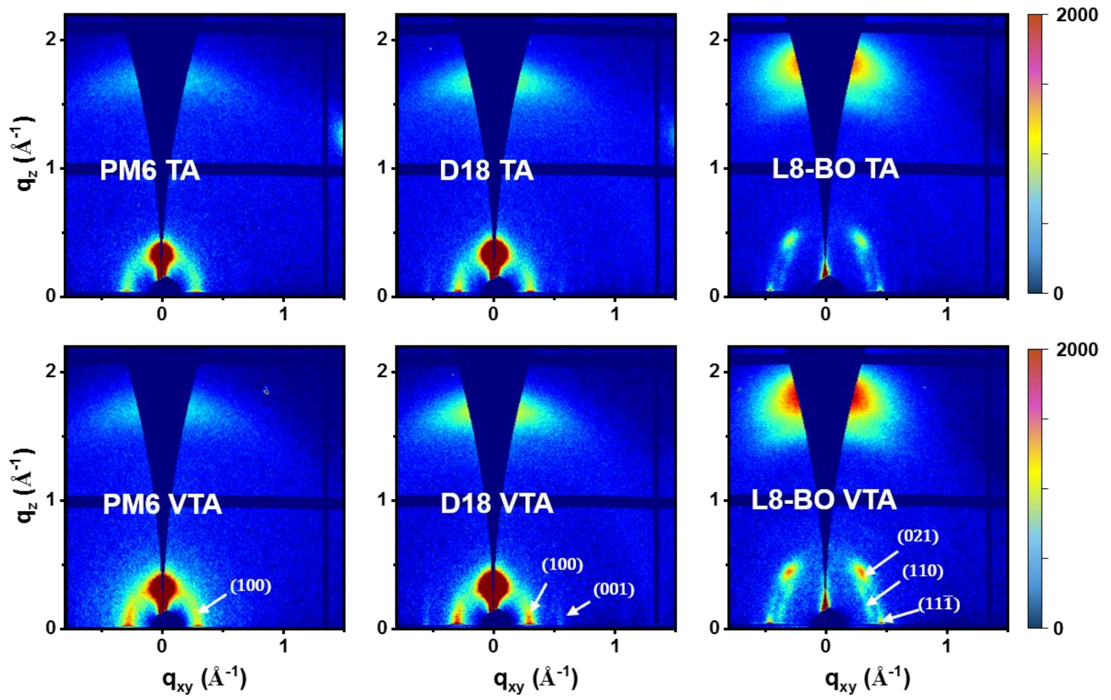

Figure S2. 2D GIWAXS patterns of PM6, D18 and L8-BO neat films fabricated upon corresponding treatments.

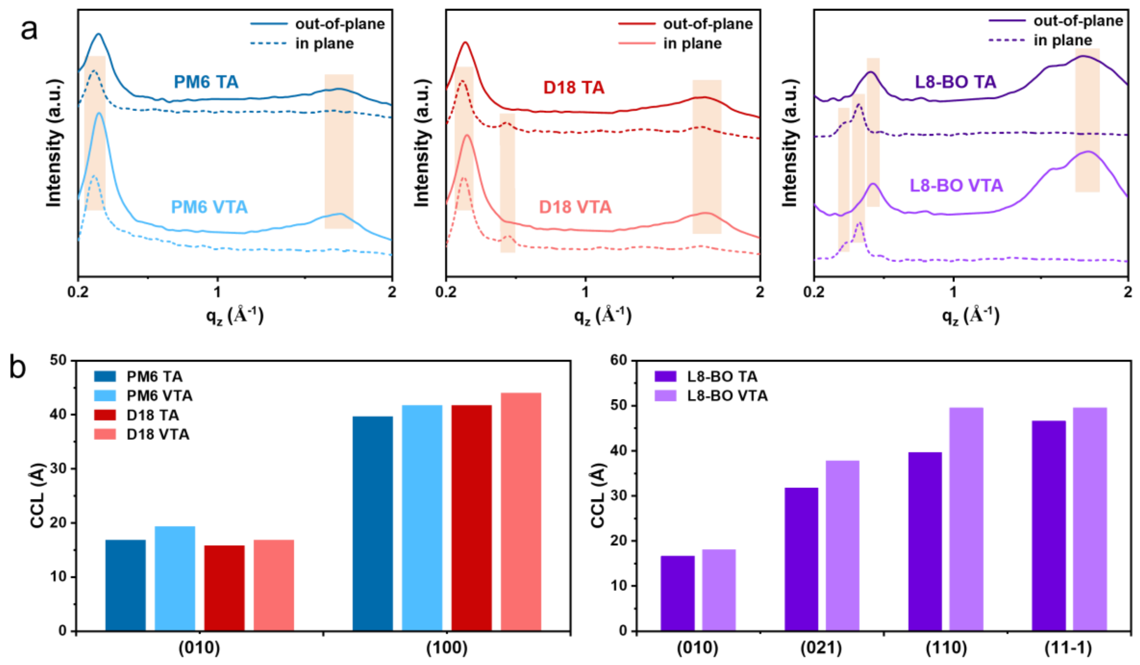

Figure S3. (a) 1D GIWAXS profiles and (b) coherence lengths of neat films processed with corresponding treatments.

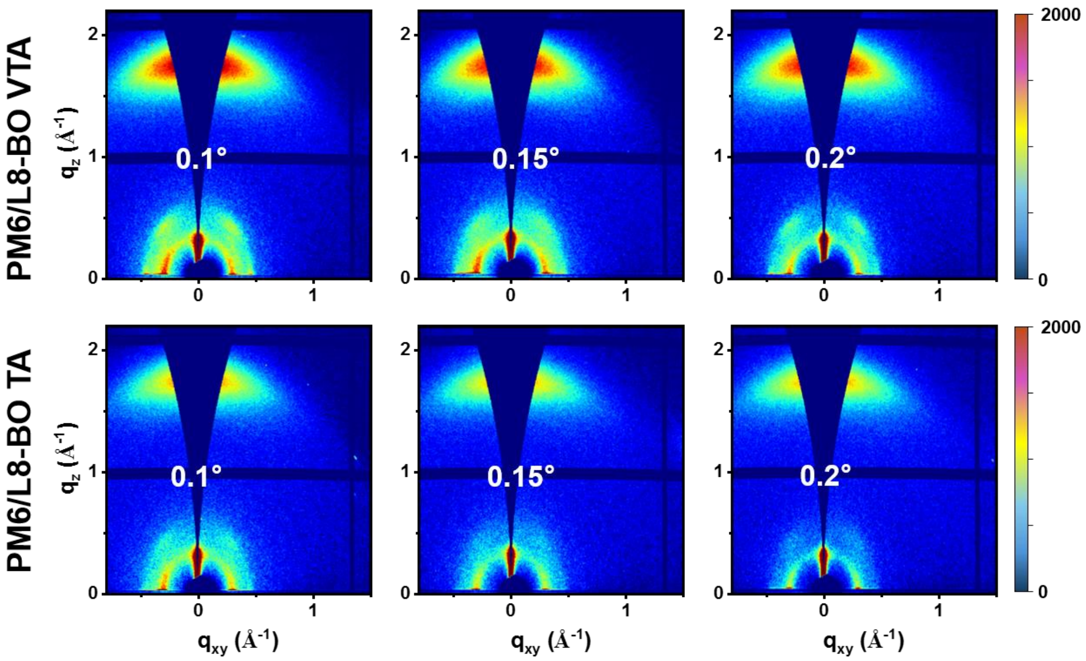

Figure S4. Angle-dependent 2D GIWAXS patterns of PM6/L8-BO films fabricated upon corresponding treatments.

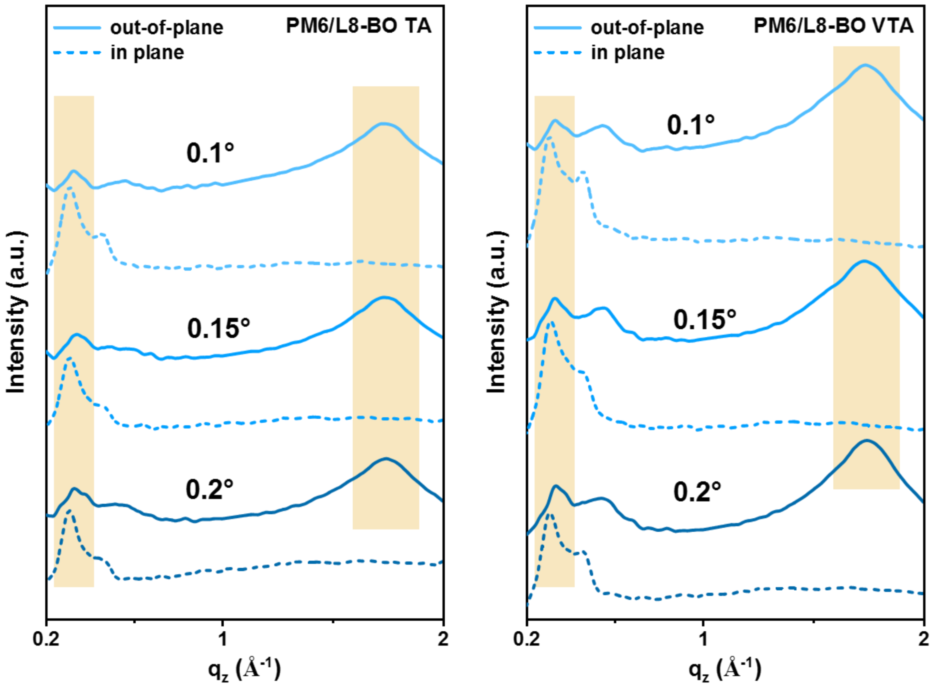

Figure S5. Angle-dependent 1D GISAXS profiles of PM6/L8-BO films processed with corresponding treatments.

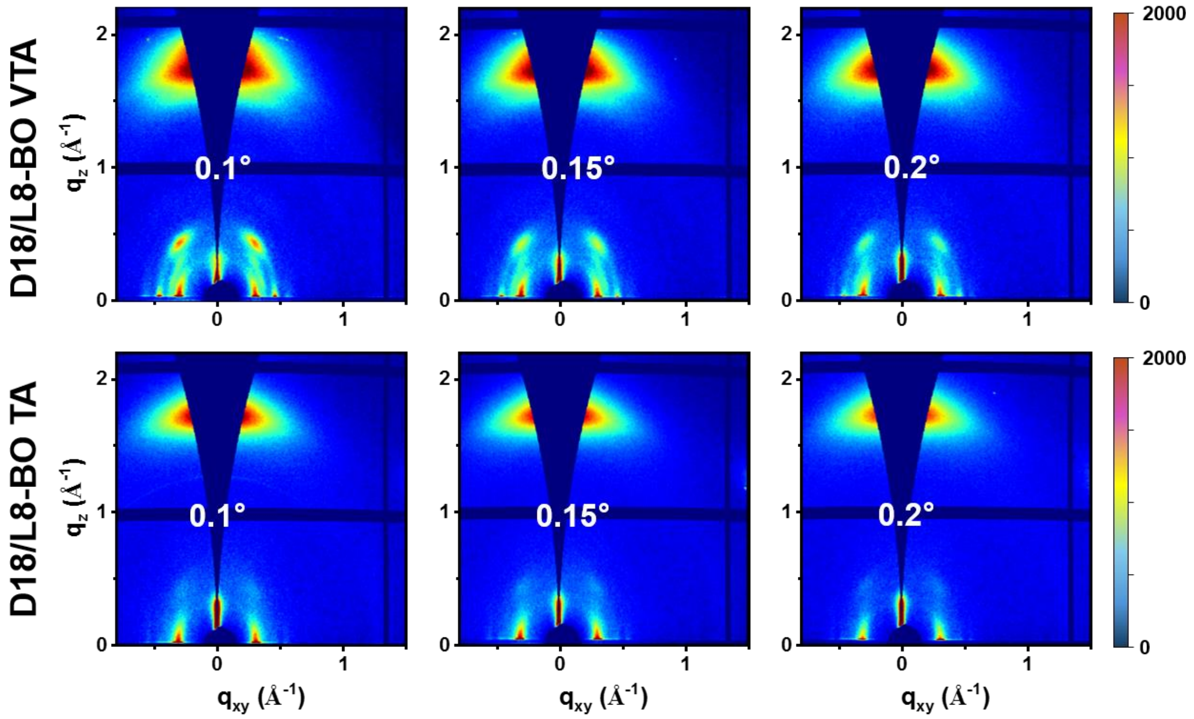

Figure S6. Angle-dependent 2D GIWAXS patterns of D18/L8-BO films fabricated upon corresponding treatments.

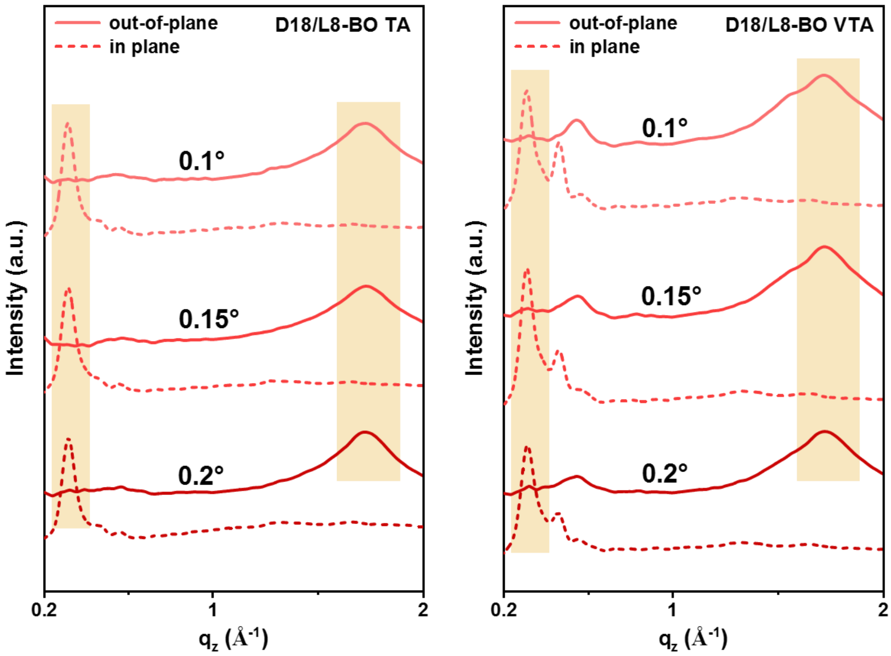

Figure S7. Angle-dependent 1D GISAXS profiles of D18/L8-BO films processed with corresponding treatments.

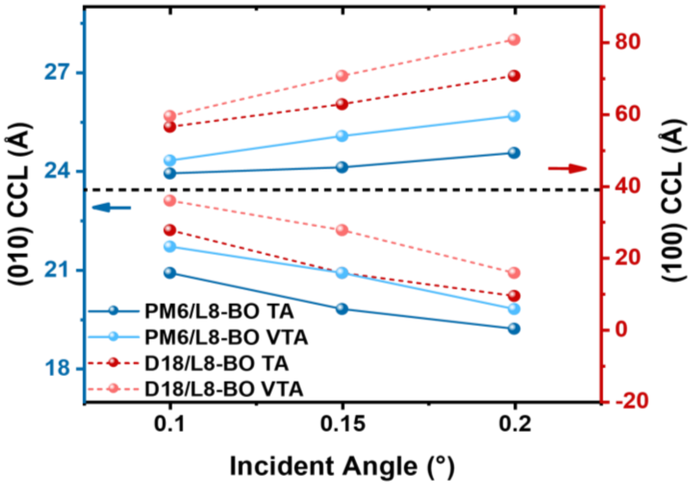

Figure S8. CCL of (010) and (100) diffraction in corresponding films versus varied incident angles.

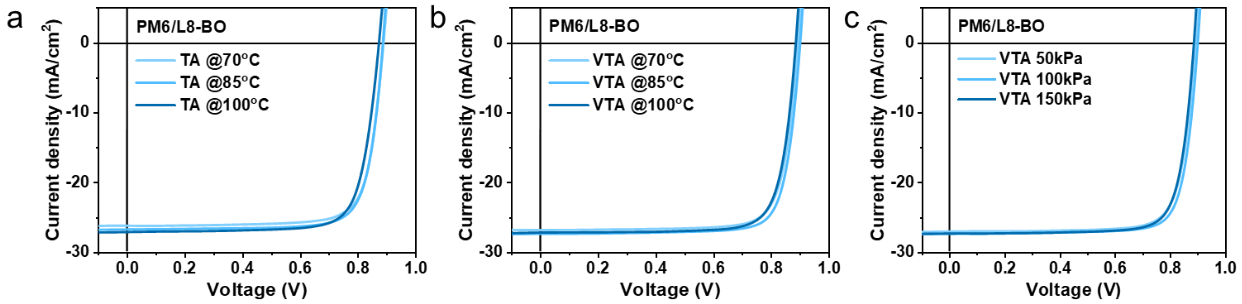

Figure S9. *J-V* curves of PM6/L8-BO OSCs fabricated with different temperature and pressure.

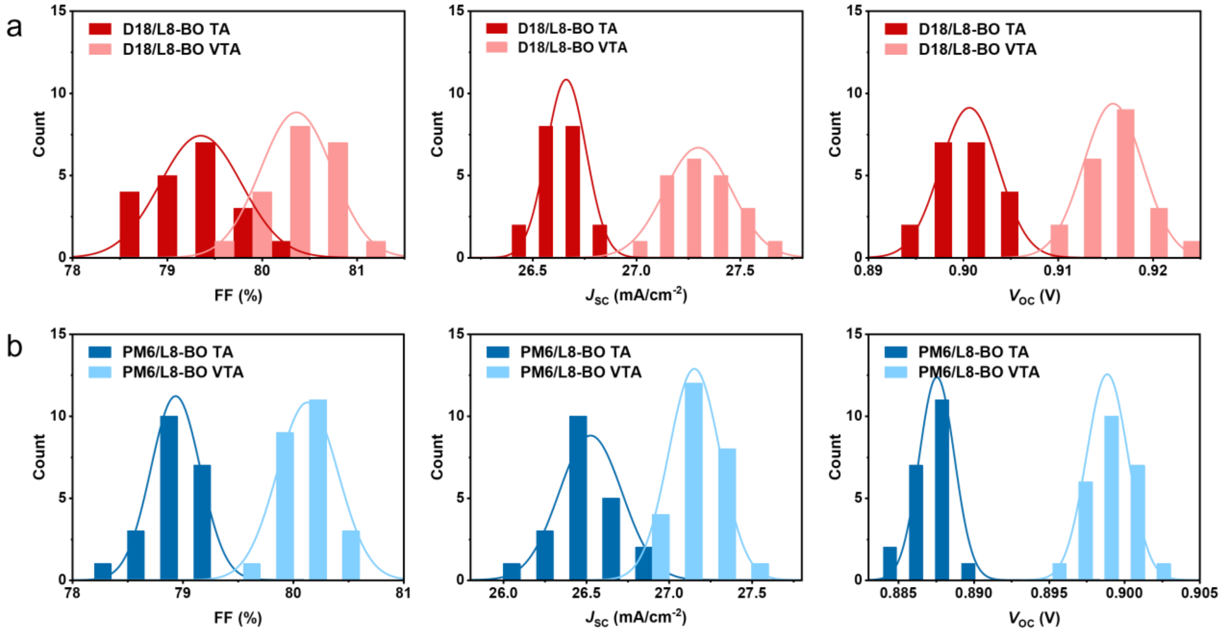

Figure S10. Histogram of photovoltaic properties counts from 20 devices for (a) D18/L8-BO and (b) PM6/L8-BO devices with corresponding treatments.

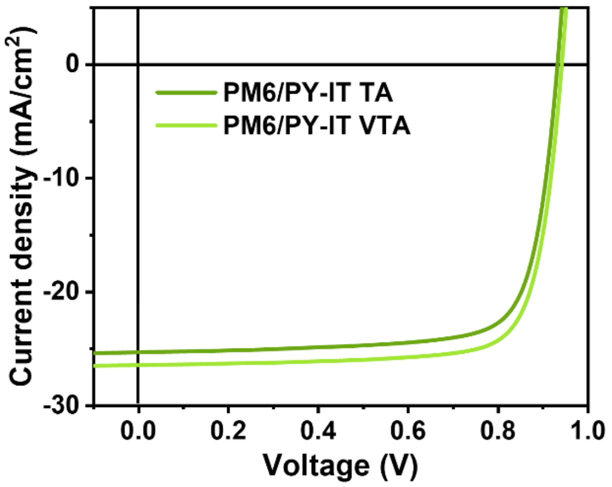

Figure S11. *J-V* curves of PM6/PY-IT OSCs fabricated with TA and VTA treatments.

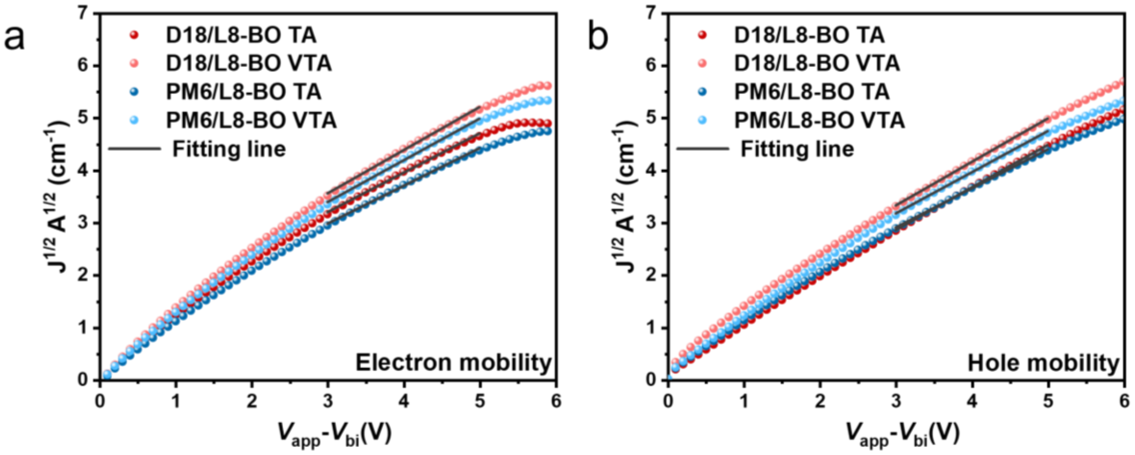

Figure S12. (a) Electron and (b) hole mobilities of OSCs processed with corresponding treatments.

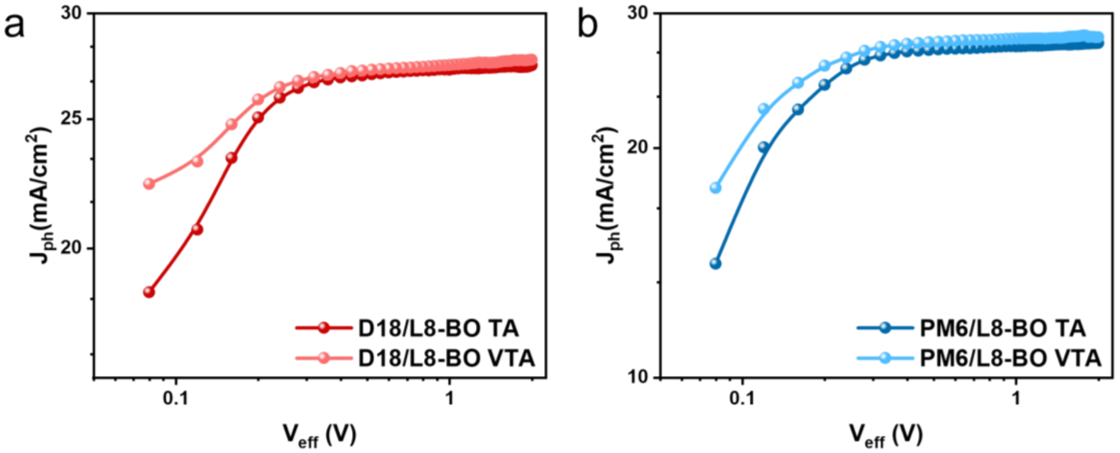

Figure S13. Photocurrent density (J_{ph}) versus effective voltage (V_{eff}) curves of (a) D18/L8-BO and (b) PM6/L8-BO OSCs with corresponding treatments.

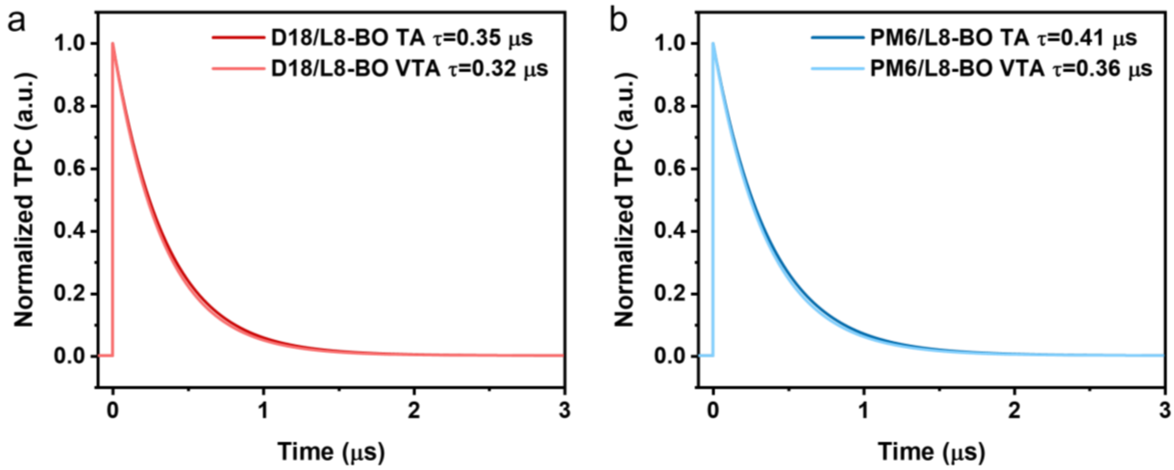

Figure S14. Normalized TPC curves of (a) D18/L8-BO and (b) PM6/L8-BO OSCs with corresponding treatments.

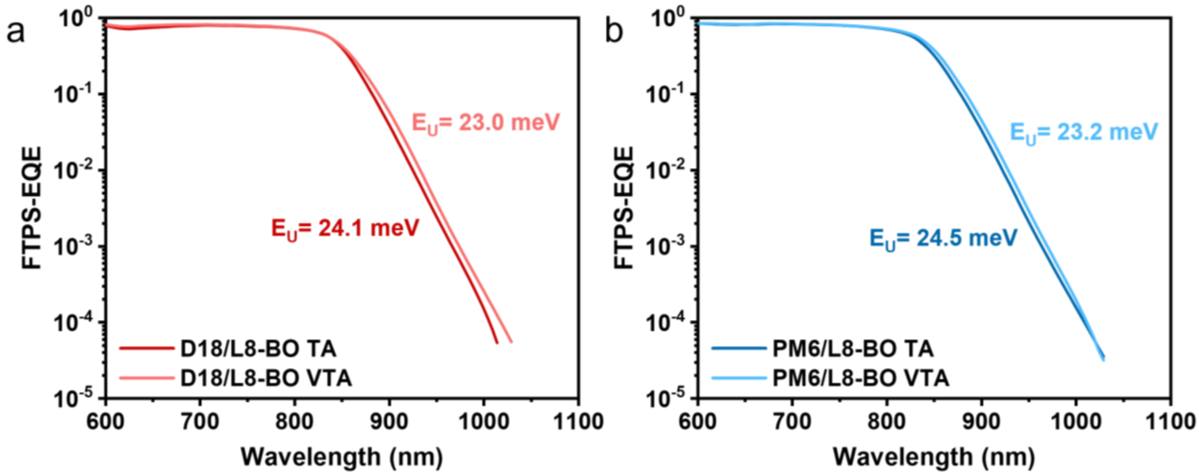

Figure S15. Fourier-transform photocurrent spectroscopy (FTPS-EQE) spectra of (a) D18/L8-BO and (b) PM6/L8-BO OSCs with corresponding treatments.

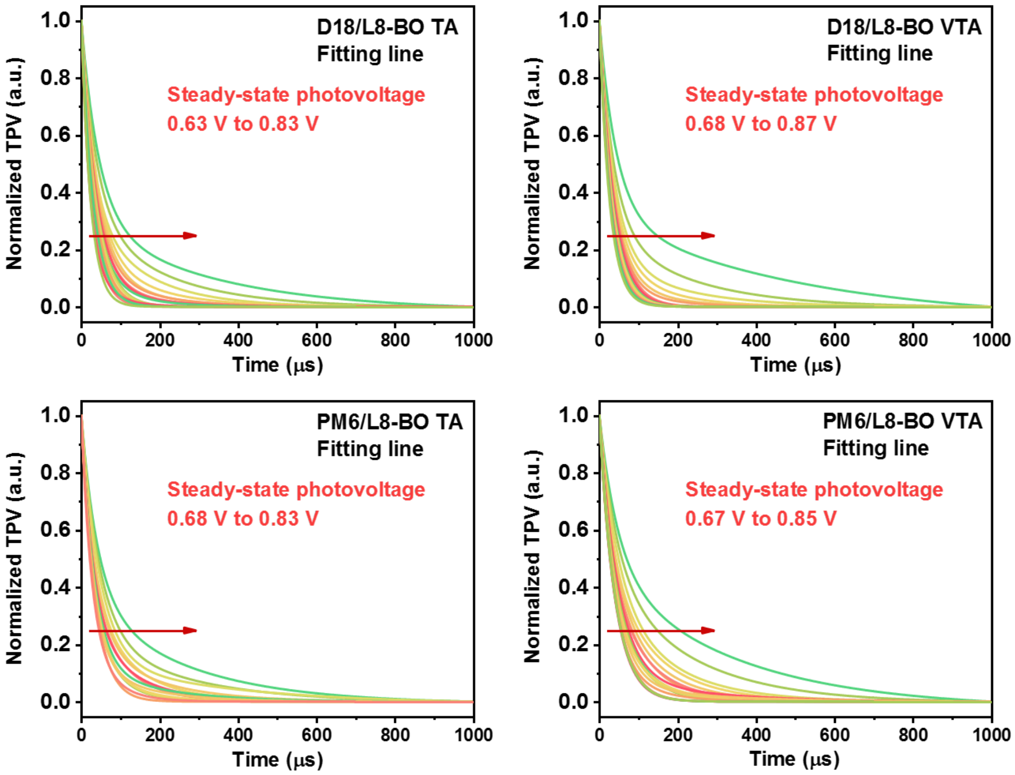

Figure S16. Voltage decay lifetime values of solar cells derived from the transient signals.

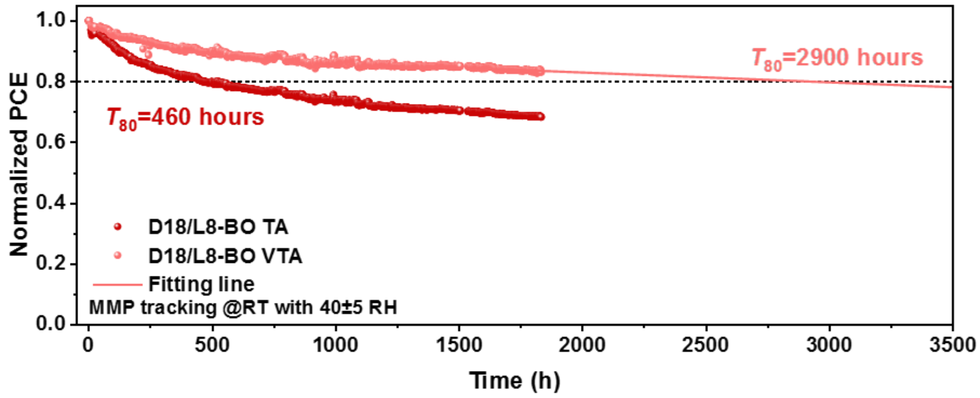

Figure S17. Maximum power point (MPP) tracking of normalized PCE for D18/L8-BO OSCs with corresponding treatments under ISOS-L-1 (one-sun illumination, 25 ± 5°C, 40 ± 5 % RH) protocol.

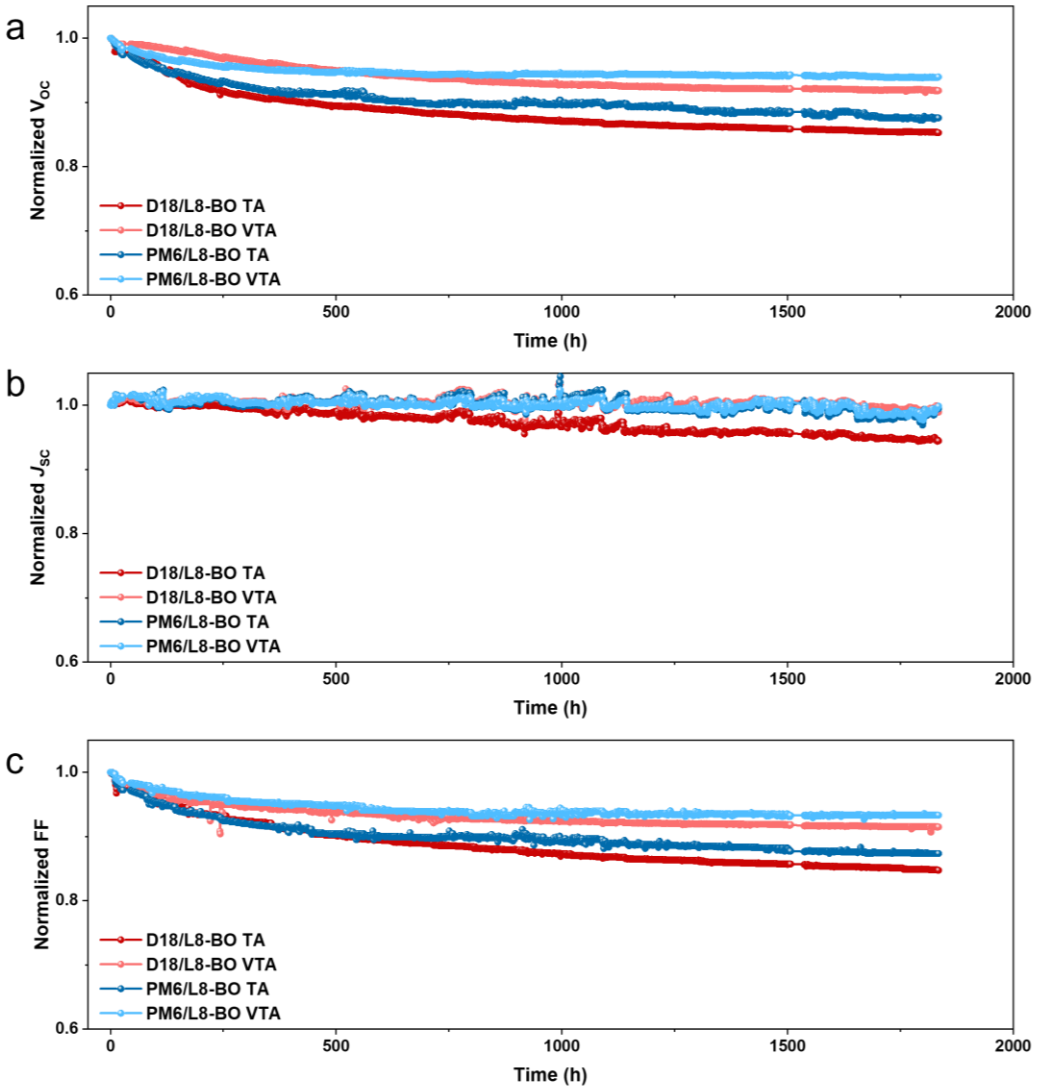

Figure S18. MPP tracking of normalized (a) V_{OC}, (b) J_{SC} and (c) FF for D18/L8-BO and PM6/L8-BO OSCs with corresponding treatments under ISOS-L-1 protocol.

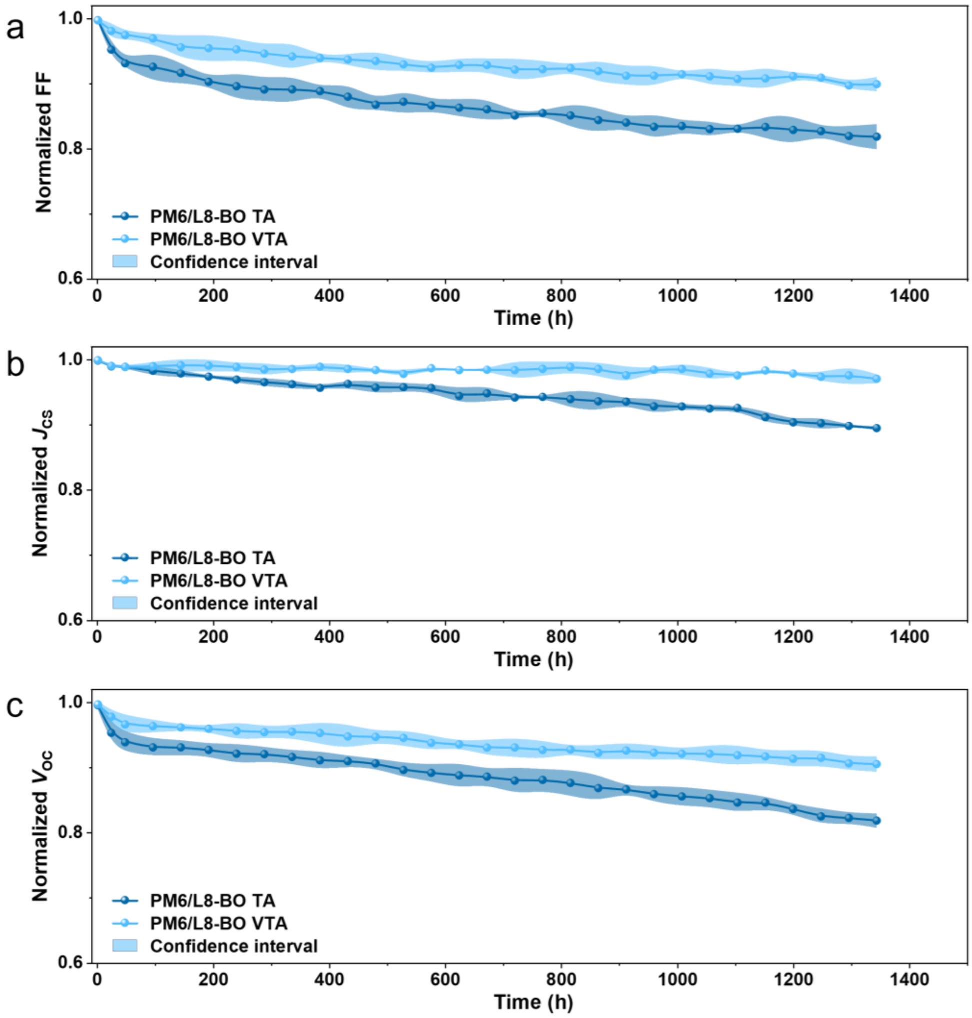

Figure S19. Normalized (a) FF, (b) J_{SC} and (c) V_{OC} for PM6/L8-BO OSCs with corresponding treatments under ISOS-L-3 (one-sun illumination, 65 ± 5°C, 65 ± 10% RH) protocol.

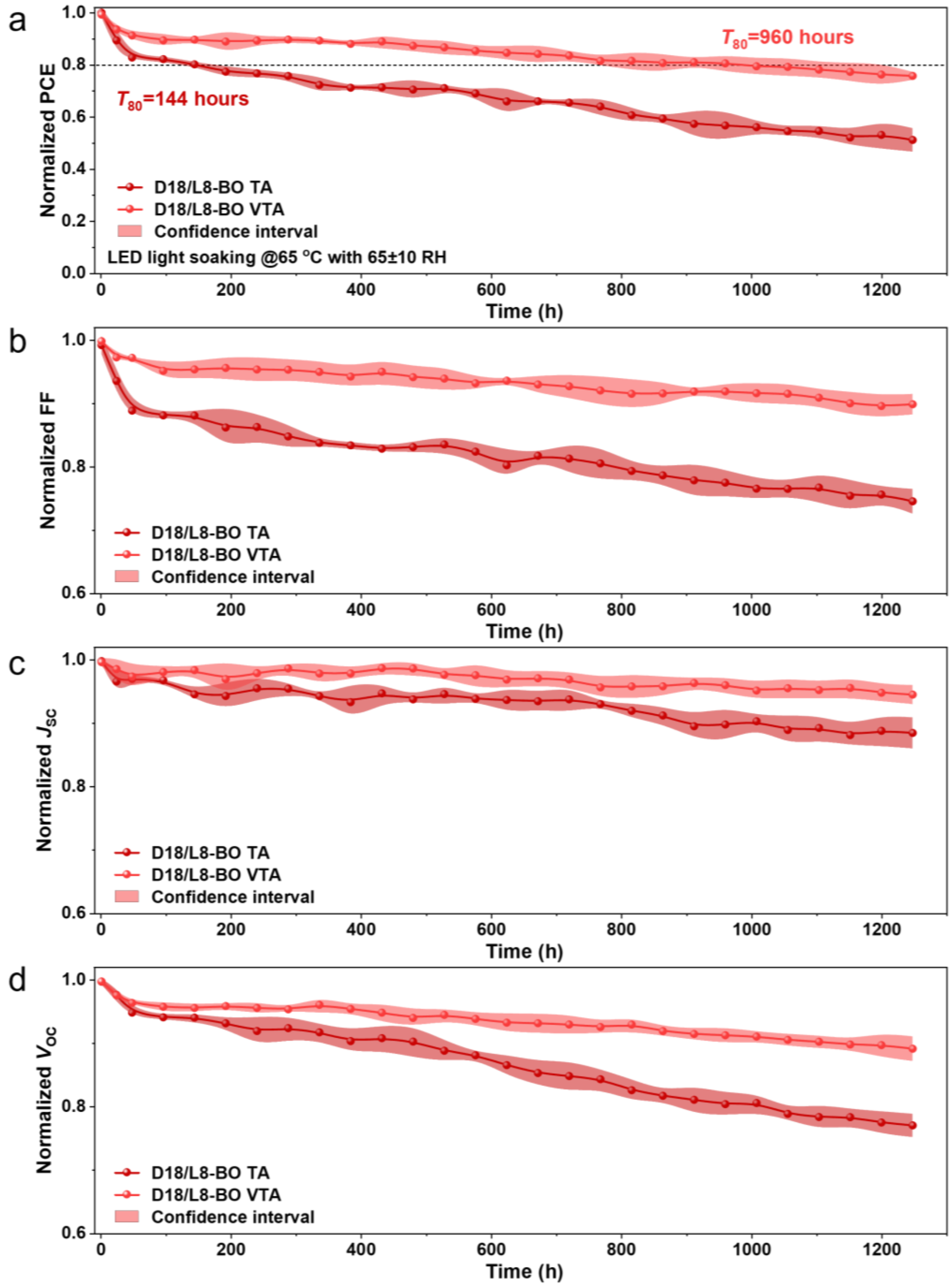

Figure S20. Normalized (a) PCE, (b) FF, (c) J_{SC} and (d) V_{OC} for D18/L8-BO OSCs with corresponding treatments under ISOS-L-3 protocol.

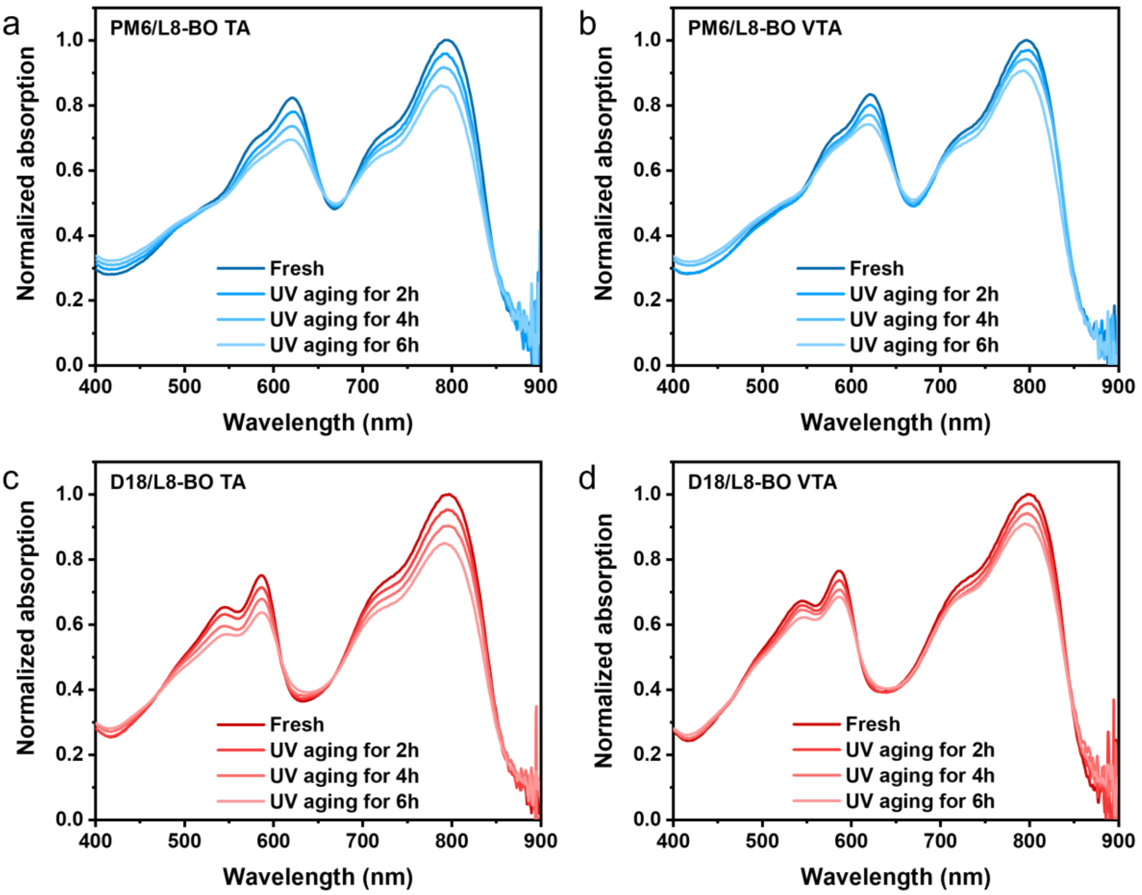

Figure S21. Absorption spectra of related films before and after UV aging for 6 hours in air.

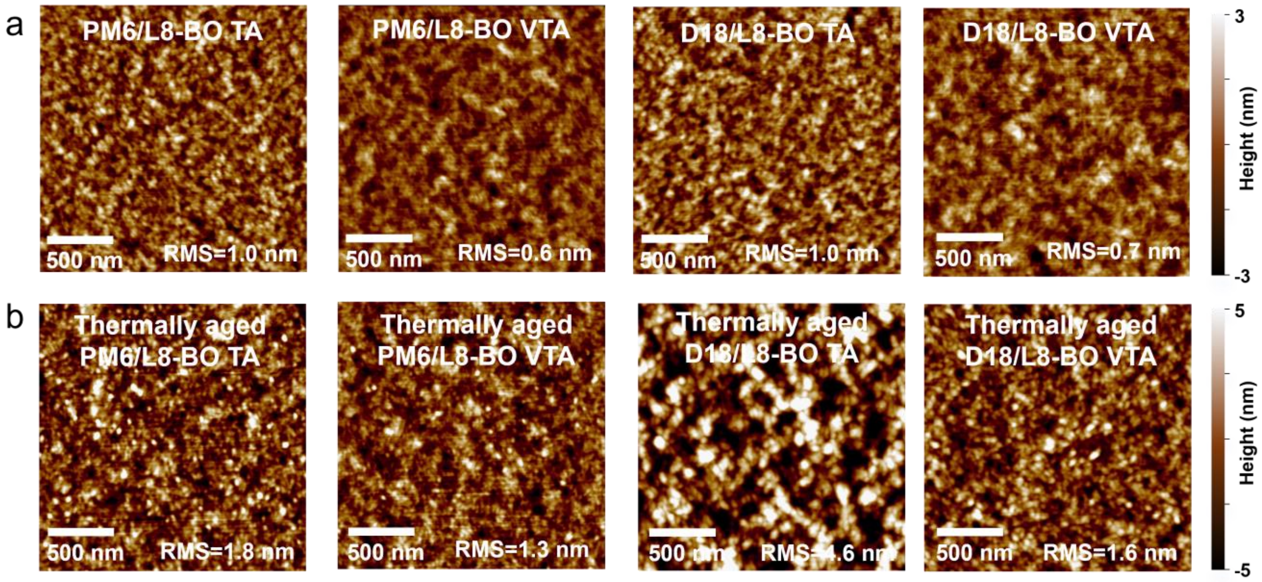

Figure S22. AFM height images of PM6/L8-BO and D18/L8-BO films (a) before and (b) after thermal aging for 4 hours at 150°C in air.

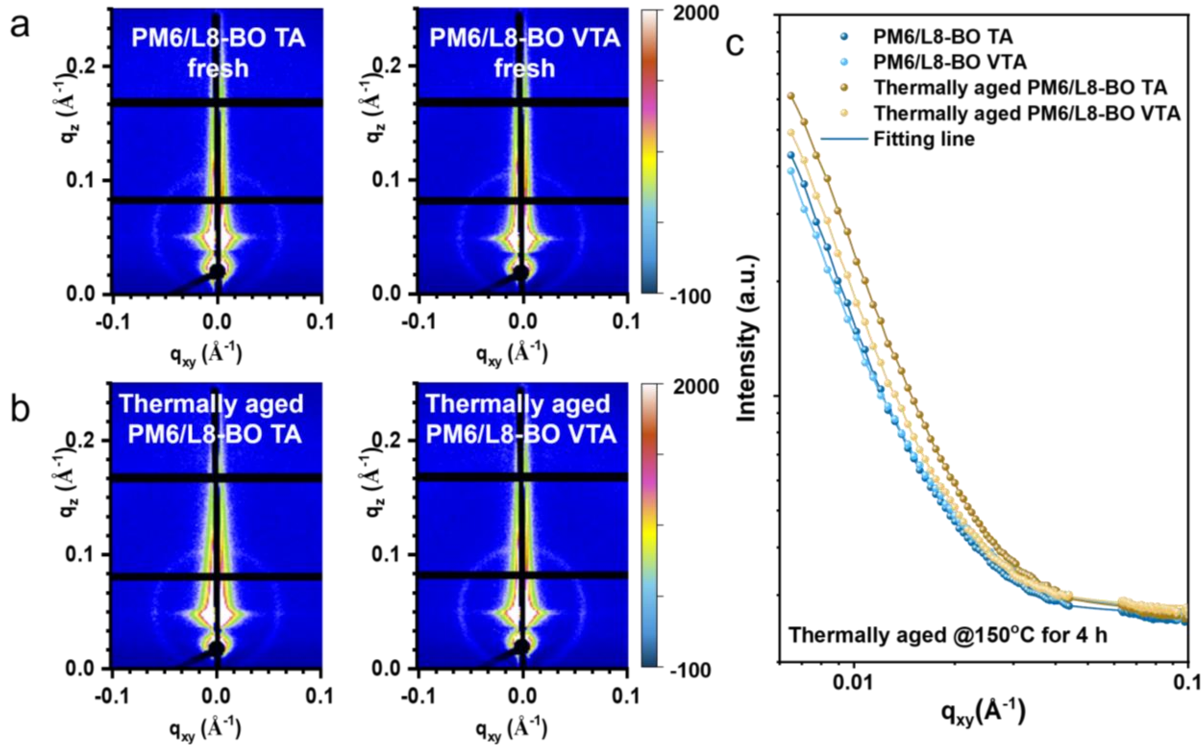

Figure S23. 2D GISAXS patterns of PM6/L8-BO films (a) before and (b) after thermal aging for 4 hours at 150°C in air. (c) 2D GISAXS profiles before and after thermal aging.

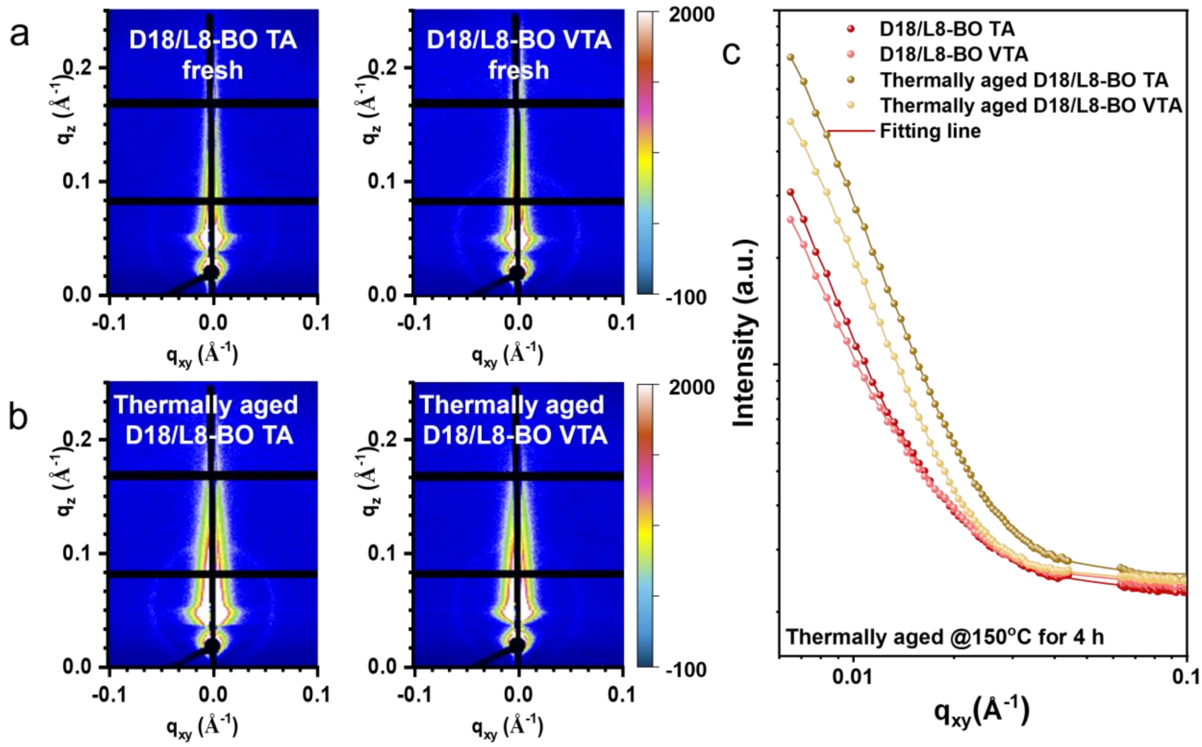

Figure S24. 2D GISAXS patterns of D18/L8-BO films (a) before and (b) after thermal aging for 4 hours at 150°C in air. (c) 2D GISAXS profiles before and after thermal aging.

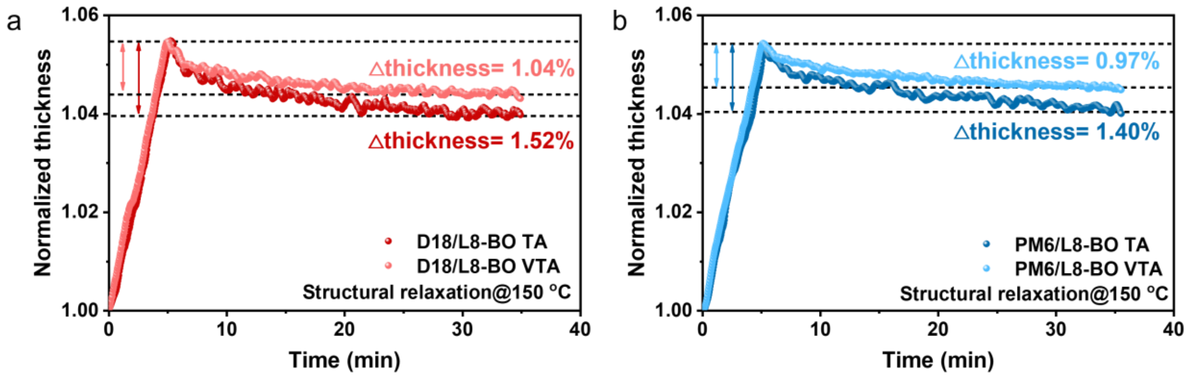

Figure S25. Thermal expansion of (a) D18/L8-BO and (b) PM6/L8-BO films for corresponding treatments during and structural relaxation.

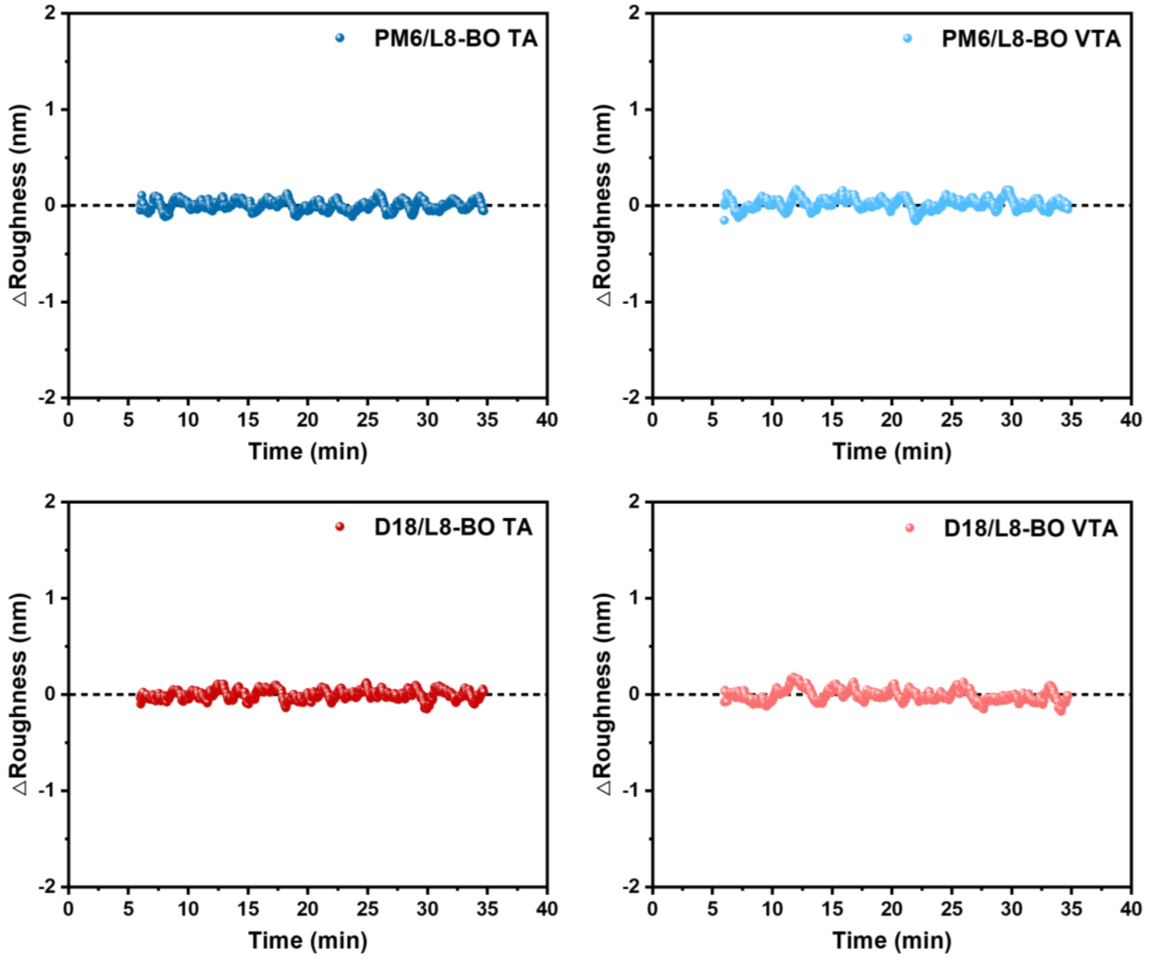

Figure S26. Roughness of related PM6/L8-BO and D18/L8-BO films during iso-hold.

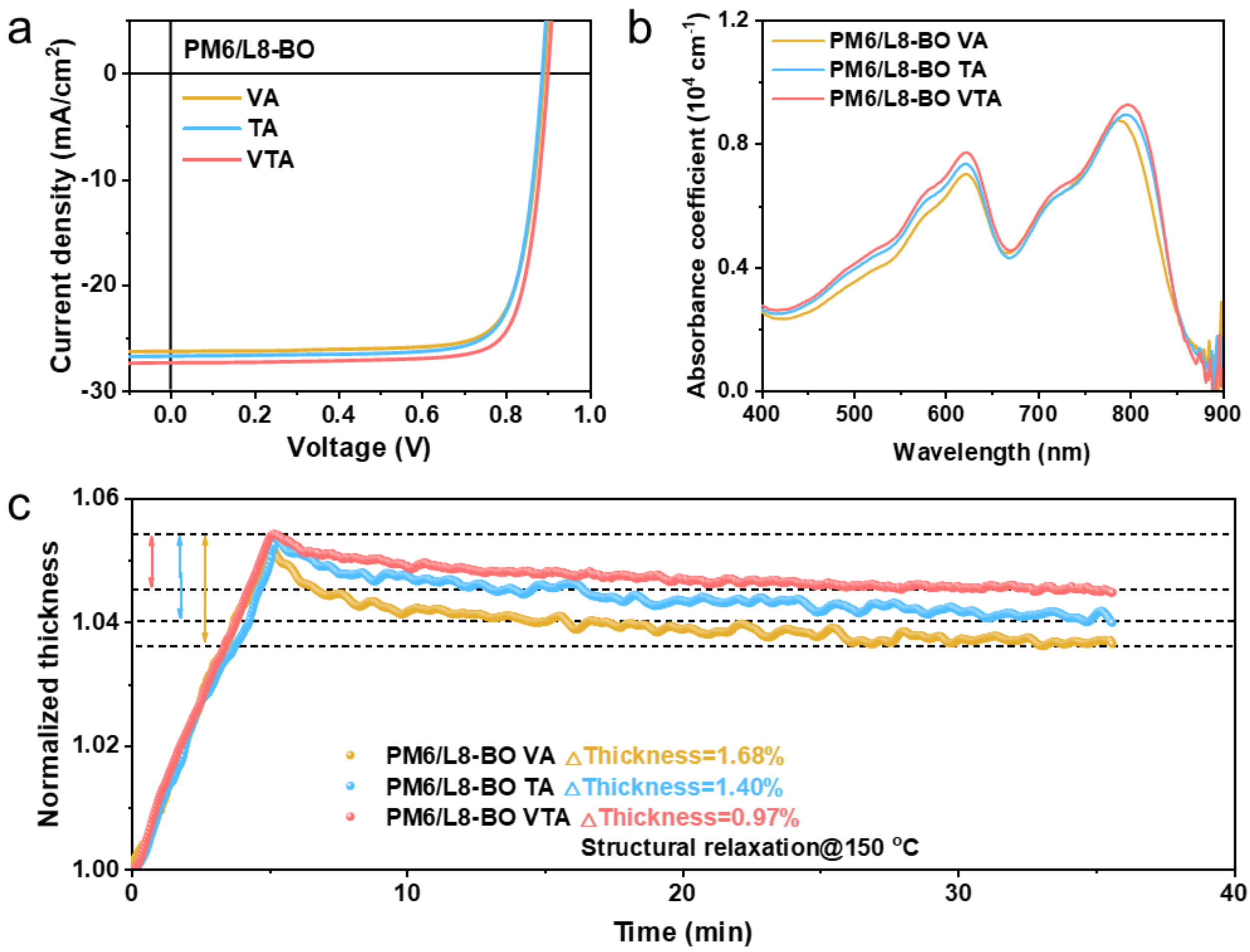

Figure S27. (a) *J-V* curves of PM6/L8-BO OSCs, with their (b) absorption coefficient and structural relaxation behavior upon different treatments.

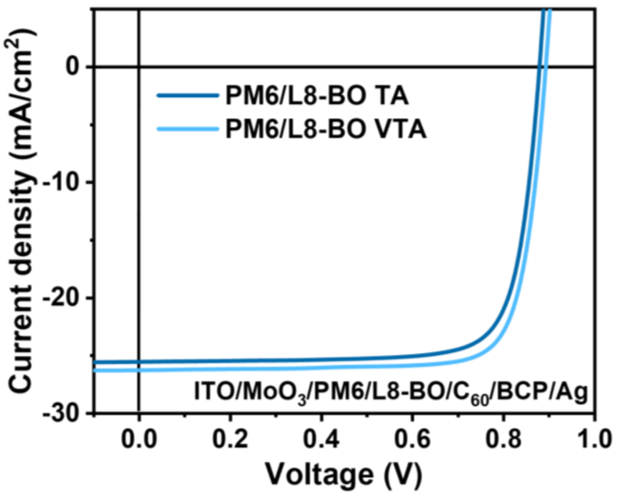

Figure S28. *J-V* curves of ITO/MoO_3/PM6/L8-BO/C_{60}/BCP/Ag devices.

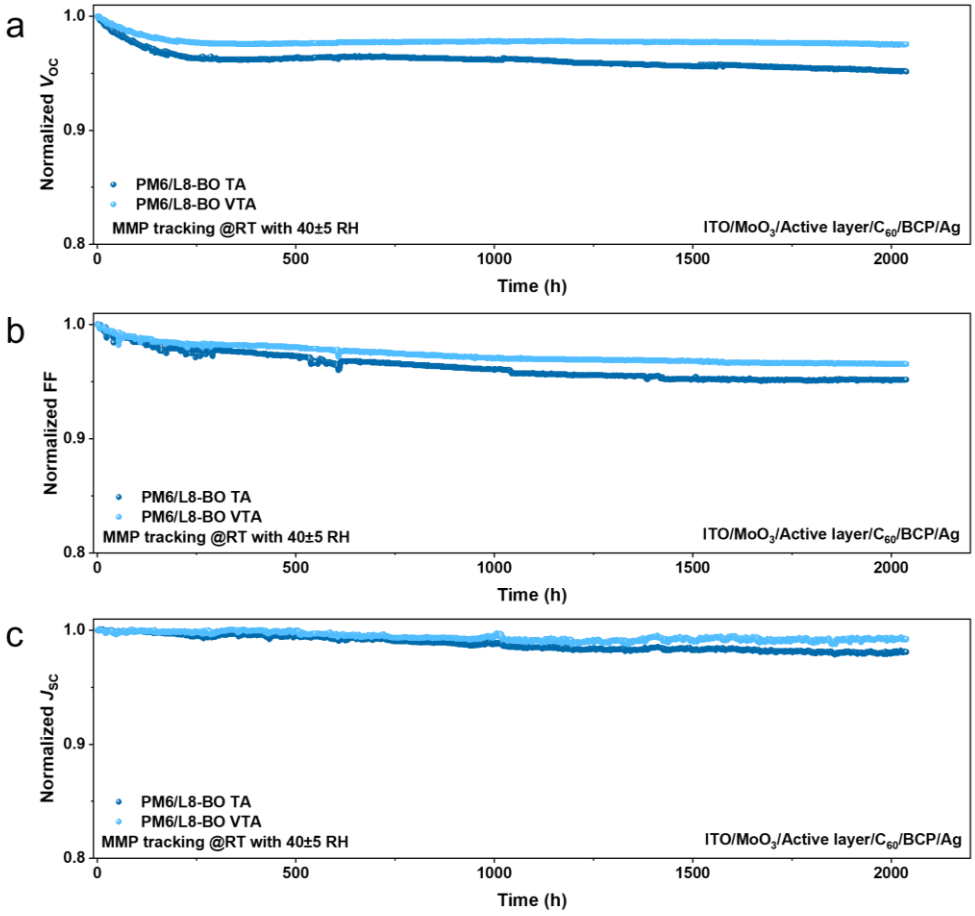

Figure S29. MPP tracking of normalized (a) V_{OC}, (b) J_{SC} and (c) FF for PM6/L8-BO OSCs with corresponding treatments under ISOS-L-1 protocol. The device structure is ITO/MoO_3/PM6/L8-BO/C_{60}/BCP/Ag.

Table S1. Detailed GIWAXS data of pseudo-bulk heterojunction (p-BHJ) films.

System	(010) π-π stacking in OOP			(100) Lamellar stacking in IP		
	q (Å^{-1})	*d*-spacing (Å)	CCL (Å)	*q* (Å^{-1})	*d*-spacing (Å)	CCL (Å)
PM6/L8-BO TA	1.74	3.61	20.9	0.30	20.9	43.5
PM6/L8-BO VTA	1.75	3.59	21.7	0.31	20.3	47.1
D18/L8-BO TA	1.73	3.63	22.2	0.31	20.3	56.5
D18/L8-BO VTA	1.74	3.61	23.1	0.31	20.3	59.5

Table S2. Detailed GIWAXS data of PM6 and D18 neat films.

System	(010) π-π stacking in OOP			(100) Lamellar stacking in IP		
	q (Å^{-1})	d-spacing (Å)	CCL (Å)	q (Å^{-1})	d-spacing (Å)	CCL (Å)
PM6 TA	1.70	3.70	16.9	0.29	21.7	35.3
PM6 VTA	1.71	3.67	17.7	0.30	20.9	40.4
D18 TA	1.69	3.72	17.1	0.30	21.7	37.7
D18 VTA	1.69	3.72	18.2	0.31	20.3	41.9

Table S3. Detailed GIWAXS data of L8-BO neat films in out-of-plane (OOP).

System	(010) π-π stacking in OOP			(021) Peak in OOP		
	q (Å^{-1})	d-spacing (Å)	CCL (Å)	q (Å^{-1})	d-spacing (Å)	CCL (Å)
L8-BO TA	1.74	3.61	16.6	0.52	12.1	31.7
L8-BO VTA	1.76	3.57	18.0	0.53	11.9	37.7

Table S4. Detailed GIWAXS data of L8-BO neat films in in-plane (IP).

System	(110) Peak in IP			(11-1) Peak in IP		
	q (Å^{-1})	d-spacing (Å)	CCL (Å)	q (Å^{-1})	d-spacing (Å)	CCL (Å)
L8-BO TA	0.39	16.1	39.6	0.46	13.7	46.6
L8-BO VTA	0.39	16.1	49.5	0.46	13.7	49.5

Table S5. Detailed angle-dependent GIWAXS data of p-BHJ films in (021) diffraction peak.

Incident angle (°)	System	q (Å^{-1})	d-spacing (Å)	CCL (Å)
0.1	D18/L8-BO TA	0.52	12.1	45.2
	D18/L8-BO VTA	0.54	11.6	49.2
0.15	D18/L8-BO TA	0.52	12.1	41.9
	D18/L8-BO VTA	0.53	11.9	47.1
0.2	D18/L8-BO TA	0.52	12.1	40.4
	D18/L8-BO VTA	0.53	11.9	43.5

Table S6. Detailed angle-dependent GIWAXS data of p-BHJ films in (001) diffraction peak.

Incident angle (°)	System	q (Å^{-1})	d-spacing (Å)	CCL (Å)
0.1	D18/L8-BO TA	0.54	11.6	53.9
	D18/L8-BO VTA	0.55	11.4	53.9
0.15	D18/L8-BO TA	0.54	11.6	59.5
	D18/L8-BO VTA	0.55	11.4	62.8
0.2	D18/L8-BO TA	0.54	11.6	62.8
	D18/L8-BO VTA	0.56	11.2	70.7

Table S7. Detailed angle-dependent GIWAXS data of p-BHJ films in (010) diffraction peak.

Incident angle (°)	System	q (Å^{-1})	d-spacing (Å)	CCL (Å)
0.1	PM6/L8-BO TA	1.74	3.61	20.9
	PM6/L8-BO VTA	1.75	3.59	21.7
	D18/L8-BO TA	1.73	3.63	22.2
	D18/L8-BO VTA	1.74	3.61	23.1
0.15	PM6/L8-BO TA	1.74	3.61	19.8
	PM6/L8-BO VTA	1.75	3.59	20.9
	D18/L8-BO TA	1.73	3.63	20.9
	D18/L8-BO VTA	1.74	3.61	22.2
0.2	PM6/L8-BO TA	1.74	3.61	19.2
	PM6/L8-BO VTA	1.75	3.59	20.2
	D18/L8-BO TA	1.73	3.63	19.8
	D18/L8-BO VTA	1.74	3.61	20.9

Table S8. Detailed angle-dependent GIWAXS data of p-BHJ films in (100) diffraction peak.

Incident angle (°)	System	q (Å^{-1})	d-spacing (Å)	CCL (Å)
0.1	PM6/L8-BO TA	0.30	20.9	43.5
	PM6/L8-BO VTA	0.31	20.3	47.1
	D18/L8-BO TA	0.31	20.3	56.5
	D18/L8-BO VTA	0.31	20.3	59.5
0.15	PM6/L8-BO TA	0.30	20.9	45.2
	PM6/L8-BO VTA	0.31	20.3	53.9
	D18/L8-BO TA	0.31	20.3	62.8
	D18/L8-BO VTA	0.31	20.3	70.7
0.2	PM6/L8-BO TA	0.30	20.9	49.2
	PM6/L8-BO VTA	0.31	20.3	59.5
	D18/L8-BO TA	0.31	20.3	70.7
	D18/L8-BO VTA	0.31	20.3	80.8

Table S9. Photovoltaic parameters of PM6/L8-BO devices with different annealing temperature.

Devices	Temperature	PCE (%)	FF (%)	J_{SC} (mA·cm^{-2})	V_{OC} (V)
PM6/L8-BO TA	70°C	18.3 (18.1±0.1)	78.6 (78.3±0.2)	26.2 (26.1±0.1)	0.890 (0.888±0.001)
	85°C	18.9 (18.6±0.2)	79.4 (78.9±0.3)	26.9 (26.5±0.2)	0.889 (0.888±0.001)
	100°C	18.6 (18.4±0.1)	78.1 (77.9±0.1)	27.1 (27.0±0.1)	0.875 (0.874±0.001)
PM6/L8-BO VTA	70°C	19.2 (18.9±0.2)	79.8 (79.7±0.2)	26.8 (26.6±0.2)	0.896 (0.893±0.001)
	85°C	20.0 (19.6±0.2)	80.9 (80.2±0.3)	27.5 (27.2±0.2)	0.902 (0.899±0.002)
	100°C	19.2 (19.0±0.1)	79.6 (79.4±0.1)	27.2 (27.0±0.1)	0.889 (0.887±0.001)

Table S10. Photovoltaic parameters of VTA-treated PM6/L8-BO devices with different pressure.

Devices	Vacuum degree	PCE (%)	FF (%)	JSC (mA·cm-2)	VOC (V)
PM6/L8-BO VTA	50 kPa	19.3 (19.1±0.1)	79.9 (79.5±0.2)	27.1 (26.9±0.1)	0.894 (0.893±0.001)
	100 kPa	20.0 (19.6±0.2)	80.9(80.2±0.3)	27.5 (27.2±0.2)	0.902 (0.899±0.002)
	150 kPa	19.4 (19.1±0.2)	80.0 (79.7±0.2)	27.3 (27.0±0.2)	0.889 (0.887±0.001)

Table S11. Photovoltaic parameters of PM6/PY-IT OSCs.

Devices	PCE (%)	FF (%)	JSC (mA·cm-2)	VOC (V)
PM6/PY-IT TA	18.4 (18.2±0.2)	77.5 (77.2±0.2)	25.4 (25.2±0.2)	0.936 (0.934±0.001)
PM6/PY-IT VTA	19.5 (19.3±0.2)	78.3 (78.1±0.2)	26.6 (26.3±0.2)	0.945 (0.943±0.001)

Table S12. Hole and electron mobilities of OSCs.

System	Hole mobility (μ_h) ($10^{-3}cm^{-1}V^{-1}s^{-1}$)	Electron mobility (μ_e) ($10^{-3}cm^{-1}V^{-1}s^{-1}$)	μ_h/μ_e
D18/L8-BO TA	3.34	3.66	0.91
D18/L8-BO VTA	4.22	4.29	0.98
PM6/L8-BO TA	3.07	3.30	0.93
PM6/L8-BO VTA	3.76	3.91	0.96

Table S13. The parameters of exciton dissociation and charge collection efficiency of OSCs.

System	P_{diss} [%]	P_{coll} [%]
D18/L8-BO TA	98.8	69.6
D18/L8-BO VTA	99.3	80.7
PM6/L8-BO TA	98.9	74.0
PM6/L8-BO VTA	99.5	80.6

Table S14. Detailed energy losses of PM6/L8-BO and D18/L8-BO OSCs.

System	E_g (eV)	E_{rad} (eV)	E_{loss} (eV)	ΔE_{nr} (eV)
D18/L8-BO TA	1.456	1.120	0.551	0.215
D18/L8-BO VTA	1.454	1.117	0.532	0.195
PM6/L8-BO TA	1.442	1.118	0.553	0.229
PM6/L8-BO VTA	1.440	1.114	0.538	0.212

Table S15. Fitting parameters of 1D GISAXS profiles for PM6/L8-BO films before and after thermal aging, where ϕ refers to the relative volume fraction of acceptor crystallites, ξ is the average correlation length of donor, η and D represent the correlation length and fractal dimension of the acceptor crystalline domain, and $2R_g$ is regarded as the size of the acceptor crystalline domain.

Films	ϕ(%)	ξ (nm)	η (nm)	D	$2R_g$ (nm)
PM6/L8-BO TA	30.6	22.5	8.1	2.5	33.9
PM6/L8-BO VTA	35.3	26.6	8.9	2.9	42.3
Thermally aged PM6/L8-BO TA	44.1	41.8	14.8	3.2	76.7
Thermally aged PM6/L8-BO VTA	42.9	33.7	12.3	3.1	62.0

Table S16. Fitting parameters of 1D GISAXS profiles for D18/L8-BO films before and after thermal aging.

Films	ϕ(%)	ξ (nm)	η (nm)	D	$2R_g$ (nm)
D18/L8-BO TA	36.5	29.9	9.2	2.7	41.1
D18/L8-BO VTA	41.2	33.7	11.6	3	56.8
Thermally aged D18/L8-BO TA	51.7	64.2	19.1	3.4	104.5
Thermally aged D18/L8-BO VTA	46.6	49.4	16.2	3.2	84.0

Table S17. Photovoltaic parameters of PM6/L8-BO devices with different treatments.

Devices	PCE (%)	FF (%)	J_{SC} (mA·cm^{-2})	V_{OC} (V)
PM6/L8-BO VA	18.6 (18.3±0.2)	79.4 (78.9±0.3)	26.4 (26.2±0.1)	0.893 (0.892±0.001)
PM6/L8-BO TA	18.9 (18.6±0.2)	79.4 (78.9±0.3)	26.9 (26.5±0.2)	0.889 (0.888±0.001)
PM6/L8-BO VTA	20.0 (19.6±0.2)	80.9(80.2±0.3)	27.5 (27.2±0.2)	0.902 (0.899±0.002)

Table S18. Photovoltaic parameters of ITO/MoO_3/PM6/L8-BO/C_{60}/BCP/Ag devices.

Devices	PCE (%)	FF (%)	J_{SC} (mA·cm^{-2})	V_{OC} (V)
PM6/L8-BO TA	17.9 (17.6±0.2)	79.1 (79.0±0.1)	25.5 (25.2±0.2)	0.884 (0.883±0.001)
PM6/L8-BO VTA	18.9 (18.7±0.1)	80.2 (79.9±0.2)	26.5 (26.3±0.1)	0.896 (0.894±0.002)

REFERENCES

[1] Sun Y, Seo JH, Takacs CJ *et al*. Inverted Polymer Solar Cells Integrated with a Low-Temperature-Annealed Sol-Gel-Derived ZnO Film as an Electron Transport Layer. *Adv Mater* 2011; **23**: 1679.

[2] Wang J, Jiang X, Wu H *et al*. Increasing donor-acceptor spacing for reduced voltage loss in organic solar cells. *Nat Commun* 2021; **12**: 6679.

[3] Liao HC, Tsao CS., Shao YT *et al*. Bi-hierarchical nanostructures of donor–acceptor copolymer and fullerene for high efficient bulk heterojunction solar cells. *Energy Environ Sci* 2013; **6**: 1938-1948.
